# Next generation GLP-1/GIP/glucagon triple agonists normalize body weight in obese mice

**DOI:** 10.1016/j.molmet.2022.101533

**Published:** 2022-07-07

**Authors:** Patrick J. Knerr, Stephanie A. Mowery, Jonathan D. Douros, Bhavesh Premdjee, Karina Rahr Hjøllund, Yantao He, Ann Maria Kruse Hansen, Anette K. Olsen, Diego Perez-Tilve, Richard D. DiMarchi, Brian Finan

**Affiliations:** 1 Novo Nordisk Research Center Indianapolis, Indianapolis, IN, USA; 2 Novo Nordisk A/S, Måløv, Denmark; 3 Department of Pharmacology and Systems Physiology, University of Cincinnati College of Medicine, Cincinnati, OH, USA; 4 Department of Chemistry, Indiana University, Bloomington, IN, USA

**Keywords:** Obesity, Pharmacology, Triagonist, Glucagon, GLP-1, GIP

## Abstract

**Objective:**

Pharmacological strategies that engage multiple mechanisms-of-action have demonstrated synergistic benefits for metabolic disease in preclinical models. One approach, concurrent activation of the glucagon-like peptide-1 (GLP-1), glucose-dependent insulinotropic peptide (GIP), and glucagon (Gcg) receptors (i.e. triagonism), combines the anorectic and insulinotropic activities of GLP-1 and GIP with the energy expenditure effect of glucagon. While the efficacy of triagonism in preclinical models is known, the relative contribution of GcgR activation remains unassessed. This work aims to addresses that central question.

**Methods:**

Herein, we detail the design of unimolecular peptide triagonists with an empirically optimized receptor potency ratio. These optimized peptide triagonists employ a protraction strategy permitting once-weekly human dosing. Additionally, we assess the effects of these peptides on weight-reduction, food intake, glucose control, and energy expenditure in an established DIO mouse model compared to clinically relevant GLP-1R agonists (e.g. semaglutide) and dual GLP-1R/GIPR agonists (e.g. tirzepatide).

**Results:**

Optimized triagonists normalize body weight in DIO mice and enhance energy expenditure in a manner superior to that of GLP-1R mono-agonists and GLP-1R/GIPR co-agonists.

**Conclusions:**

These pre-clinical data suggest unimolecular poly-pharmacology as an effective means to target multiple mechanisms contributing to obesity and further implicate GcgR activation as the differentiating factor between incretin receptor mono- or dual-agonists and triagonists.

## Highlights

• Details the design of unimolecular peptide triagonists for GLP-1R/GIPR/GCGR.
• Optimal weight-loss is achieved when receptor potency ratio is weighted toward GCGR vs GLP-1R or GIPR.
• These agonists are protracted for once-weekly human dosing.
• Optimized triagonists normalizes body weight & enhance energy expenditure in mice.
• Efficacy of optimized triagonists is superior to GLP-1R & GLP-1R/GIPR agonists.

## 1 Introduction

The continuing clinical successes of GLP-1 receptor (GLP-1R) agonists has reinforced the substantial potential of this pharmaceutical approach to the treatment of type 2 diabetes and obesity, and these agonists remain the only class of pharmaceuticals approved for both indications [[1], [2], [3]]. The once-daily injectable GLP-1R agonist liraglutide at a dose of 3.0 mg (Saxenda) reduces body weight by an average of 5–10% in non-diabetic obese patients [4], and the recently approved 2.4 mg dose of once-weekly GLP-1R agonist semaglutide (Wegovy) increases mean weight reduction in this population to 15–20% [[5], [6], [7], [8]]. However, further enhancement of the efficacy of GLP-1R agonists alone is limited by dose-dependent gastrointestinal events [9,10], which could be mitigated with prolonged dose escalation algorithms. However, the maximal efficacy of GLP-1R agonists alone is seemingly plateaued with increased doses, at least in diabetic patients [11]. Therefore, combinatorial approaches are being pursued in order to drive additional efficacy by leveraging distinct mechanisms of weight reduction and metabolic improvement, particularly the use of peptides exhibiting activity at multiple targets [[12], [13], [14], [15], [16]].

While a variety of partners for GLP-1R agonists are currently under investigation, two candidates have emerged with recent, provocative clinical data: glucagon (Gcg) and glucose-dependent insulinotropic polypeptide (GIP). Unimolecular co-agonists of GLP-1R and the glucagon receptor (GcgR) were the first to emerge, seeking to leverage the anorectic and anti-diabetic effects of GLP-1 with the ability of Gcg to increase energy expenditure [[17], [18], [19]]. After an initial flurry of pre-clinical reports highlighting additive effects on weight reduction (for a review, see [20]), data from clinical trials of once-daily GLP-1R/GcgR co-agonists cotadutide (previously MEDI0382) [[21], [22], [23]] and SAR425899 [24,25] have generated guarded optimism over the modest effects on weight reduction (<10% after 4–6 weeks of treatment). However, it is important to note that the diabetogenic effects of GcgR agonism need to be offset by the insulinotropic effects of GLP-1, typically steering the molecular design of such co-agonists toward greater GLP-1R potency than GcgR potency by virtue of reducing the relative GcgR potency by as much as 10-fold [[26], [27], [28]]. While this selection criterion ensures safety from the standpoint of glycemic control, it also limits the weight lowering efficacy of the glucagon component, which has an inherently steep dose response curve at least in pre-clinical models. This suggests that inclusion of additional insulinotropic targets that enhance hyperglycemic buffering may facilitate more robust GcgR agonism and reciprocally allow for greater maximal weight loss.

Interest in GIP as a pharmaceutical agent has grown recently after decades of languishing in the shadows of its fellow incretin, GLP-1, though the direction to manipulate receptor function to unleash its therapeutic potential remains unsettled [[29], [30], [31]]. Indeed, agonists and antagonists of the GIP receptor (GIPR) are currently in development, both of which have paradoxically demonstrated the ability to potentiate the weight reduction of GLP-1R agonists in preclinical models [[32], [33], [34], [35], [36], [37]]. The issue is further complicated by recent data demonstrating that GIPR agonism in the CNS reduces body weight by reducing food intake while peripheral GIPR agonism acts through food intake independent means to achieve weight loss [38]. Neutralizing GIPR antibodies, which are presumably sequestered to the periphery, reduce body weight in an independent manner to food intake and modify energetic substrate utilization in vivo [33]. These data demonstrate that GIPR modulation can serve both to enhance the anorectic effects of GLP-1R agonism and facilitate additional weight loss through other putative mechanisms such as improvement in insulin sensitivity [30] and reduction in GLP-1R mediated adverse gastrointestinal events to permit increased GLP-1R engagement [39]. To date, clinical results are available for two unimolecular co-agonists of GLP-1R and GIPR: once-daily NN0090-2746 (previously RG7697 and MAR709) [40] and once-weekly tirzepatide (previously LY3298176) [41]. Clinical efficacy data are particularly compelling for tirzepatide. A phase 3 study in type 2 diabetic patients demonstrated that, when compared head-to-head against semaglutide at the 1.0 mg dose level, tirzepatide not only further improved HbA1c levels at all doses tested (5–15 mg), but the highest dose level of tirzepatide also doubled the average weight loss after 40 weeks of treatment achieved with semaglutide (−12.4 kg compared to −6.2 kg) [42].

The reinforcing incretin effects of GLP-1R and GIPR agonism on glycemic control logically seem an appropriate buffer to the hyperglycemic liability of GcgR agonism [43]. This could allow a higher ceiling for efficacy through both a wider therapeutic window for the Gcg component as well as harnessing three complementary mechanisms of weight reduction. Thus “triple agonism” of GLP-1R, GIPR, and GcgR could represent a new standard for pharmaceutical intervention in obesity [44,45]. In support of this notion, unimolecular triple agonists with time-action appropriate for once-daily clinical administration demonstrate the metabolic benefits of the three combined activities in preclinical settings [43,46].

Delineating the relative contribution of GcgR agonism to the efficacy of triple GLP-1R/GIPR/GcgR agonists remains a critical question in the field of obesity pharmacology. In this work, we sought to address this question by rationally designing peptide backbones with fatty acid-based protractors intended to permit once-weekly dosing in humans to create high potency triple agonists with variable ratios of activation between the three constituent receptors. These novel, unimolecular GLP-1R/GIPR/GcgR triple agonists display extended time-action, supporting the potential of once-weekly administration in humans. Critically, administration of these compounds reduced body weight in diet-induced obese (DIO) mice with greater potency and efficacy than could be achieved with the clinical GLP-1R mono-agonist semaglutide, the experimental GLP-1R/GIPR dual agonist tirzepatide, or GLP-1/GcgR co-agonists, and other reported triagonists without sacrificing glycemic control.

## 2 Methods

### 2.1 Peptide synthesis, purification, and analysis

Peptides were built by standard fluorenylmethoxycarbonyl (Fmoc)-based solid-phase synthesis using one of the following resins: Rink Amide-ChemMatrix resin, Rink Amide AM polystyrene resin, or PAL Amide AM resin. Automated peptide synthesis was performed using one of the following protocols: a SymphonyX peptide synthesizer (Protein Technologies) employing 20% piperidine in dimethylformamide for Fmoc deprotection and diisopropylcarbodiimide (DIC)/ethyl cyanohydroxyiminoacetate for amino acid coupling; or a 431A peptide synthesizer (Applied Biosciences) employing 20% piperidine/*N*-methyl-2-pyrrolidone for Fmoc deprotection and DIC/6-chloro-1-hydroxybenzotriazole for amino acid coupling. For peptides bearing an acylation, the lysine to be acylated was incorporated with N^ε^-4-methyltrityl (Mtt) protection and the N-terminal amino acid with N^α^-Boc protection. Following completion of the peptide backbone, Mtt deprotection was performed with 30–75% hexafluoroisopropanol in dichloromethane. The acylation was either built by the same Fmoc-based chemistry as described above or added as a single unit via a succinimidyl ester. Following completion of peptide synthesis, the peptide was cleaved from the resin by treatment with 2.5% water/2.5% triisopropylsilane in trifluoroacetic acid (TFA) for 2 h, followed by precipitation with diethyl ether and isolation by centrifugation. The precipitate was dissolved in a water containing sufficient acetonitrile to solubilize, allowed to stand at room temperature until all labile adducts decomposed, and purified by reversed-phase high-performance liquid chromatography (RP-HPLC) on a C8 or C18 column with a gradient of acetonitrile in 0.1% aqueous TFA. Relevant fractions were analyzed for identity and purity by analytical RP-HPLC with electrospray ionization mass spectrometry (Methods Table 1). Appropriate fractions were pooled and lyophilized to afford the desired peptide as a white solid.

### 2.2 Receptor activation

Cyclic AMP (cAMP) production was assessed as a proxy for receptor activation using stably transfected baby hamster kidney (BHK) cell lines expressing the DNA for one individual receptor (GLP-1R, GIPR, GcgR; human- or mouse-specific sequence) and the DNA for the firefly luciferase reporter gene linked to the cAMP response element (CRE). The cells were kept in continuous culture at 37 °C and 5% CO_2_ in Dulbecco's Modified Eagle Medium (DMEM) supplemented with 10% heat-inactivated fetal bovine serum (HI-FBS), 300 μg/mL hygromycin, and 500 μg/mL G418; for cell lines expressing GIPR, the G418 concentration was lowered to 400 μg/mL. Cells were plated in a 96-well poly-d-lysine-coated plate at 5,000 cells per well in growth media and incubated overnight at 37 °C with 5% CO_2_. After the overnight incubation, the media was removed, the plate was washed once in Dulbecco's phosphase-buffered saline (DPBS), and 50 μL of assay buffer (DMEM without phenol red, 10 mM HEPES, 1x Glutamax, 1% ovalbumin, 0.1% Pluronic F-68) was added to each well. Compounds to be tested were serially diluted 3.5-fold across the rows of a separate low-bind 96-well plate to create a 12-point dilution curve in assay buffer. Aliquots of the dilution curves were added to the cell plate in a volume of 50 μL per well resulting in final assay concentrations ranging from 1 × 10^−14^ to 1 × 10^−7^ M. The assay plate was incubated for 3 h at 37 °C and 5% CO_2_. After the incubation, the assay plate was washed once with DPBS. A 100 μL aliquot of DPBS was added to each well followed by 100 μL of steadylite plus reagent (PerkinElmer). The assay plate was covered to protect reagent from light, shaken at 250 rpm at room temperature for 30 min, and read in a microtiter plate reader. EC_50_ values were calculated using Prism software (GraphPad) with the nonlinear regression log(agonist) vs. response. A minimum of two replicates were measured for each sample.

### 2.3 Pharmacokinetics in minipigs

Studies were conducted in accordance with the Protection of Animals Act, the Act on Experiments on Animals, and the Standard Operating Procedures for Experiments on Animals at Novo Nordisk A/S. The experiments were performed under the supervision and approval of the Danish Government Animal Experiments Inspectorate and the Novo Nordisk Ethical Review Counsel. Female Göttingen minipigs (Ellegaard Göttingen Minipigs A/S), 7–14 months old and weighing 16–35 kg, were housed individually and fed once daily with a restricted SDS minipig diet (Special Diets Services). At least one week before the study, two permanent central venous catheters were implanted in the caudal vena cava of each animal. The animals were fasted for approximately 18 h before dosing and 4 h after dosing but had ad libitum access to water during the whole period. Test compounds dissolved to a concentration of 20–40 nmol/mL in a vehicle (pH 7.4) containing 0.025% polysorbate-20, 10 mM sodium phosphate, and 250 mM glycerol were administered via an intravenous injection through one catheter at a dose of 2 nmol/kg. Blood samples were collected in 8 mM aqueous EDTA for up to 14 days post dosing, preferably through the other catheter, then centrifuged at 4 °C and ∼2000g for 10 min. Plasma was isolated and immediately frozen until RP-HPLC/MS analysis. Individual plasma concentration–time profiles were analysed by a non-compartmental model in Phoenix WinNonLin software (Pharsight Inc.), and the resulting terminal half-lives were determined at the harmonic mean.

### 2.4 Pharmacodynamics in DIO mice

Studies were approved by and performed according to the guidelines of the Institutional Animal Care and Use Committee of the University of Cincinnati. Male C57BL/6J mice (Jackson Laboratories) were housed 4 per cage, exposed to a controlled 12 h/12 h light–dark cycle at room temperature (22 °C), and provided ab libitum access to water and a 58% fat, high-sugar diet (D12331, Research Diets) for 12 weeks. Mice exceeding 50 g of body weight were considered diet-induced obese (DIO) and included in studies, where they were randomized and evenly distributed to test groups (n = 8 per group) according to body weight. Test compounds dissolved at a concentration of 0.5–2 μM in a vehicle (pH 7.4) containing 0.05% polysorbate-80, 50 mM sodium phosphate, and 70 mM sodium chloride were administered subcutaneously once daily, unless otherwise noted, during the light cycle for each day of treatment at a volume of 2–5 μL per gram of body weight as necessary to achieve the desired dose. Body weight and food intake were measured immediately prior to dosing each day. The percent change in body was calculated individually for each mouse based on initial body weight prior to the first injection. For intraperitoneal (i.p.) glucose tolerance tests (IPGTT), animals were fasted for 6 h prior to the test but had access to water. Fasted blood glucose levels were measured, then mice were injected with an i.p. glucose load of 2 g/kg from a 200 mg/mL aqueous glucose solution. Tail blood glucose levels were measured 0, 15, 30, 60, 90, and 120 min following the glucose load using a handheld glucometer (Freestyle, Abbott).

### 2.5 Energy expenditure in DIO mice

Energy expenditure was assessed using a combined indirect calorimetry system (TSE Systems, Chesterfield, MO). Animals were kept in the indirect calorimetry system for 7d; d1 (0–24h) included acclimation to the new cages but no energy expenditure readings, d2 included baseline energy expenditure readings with vehicle injections, d3-7 included experimental energy expenditure readings and treatment injections. Treatment injections begin at t = 48h as indicated by the vertical dashed line and were performed every 24h as indicated by the arrows. O_2_ consumption and CO_2_ production were measured every 20 min to determine the respiratory quotient and energy expenditure using the Weir equation (EE=(3.94 x vO_2_) + (1.1 x vCO_2_)).

### 2.6 Pharmacodynamics in db/db mice

Studies were approved by and performed according to the guidelines of the Institutional Animal Care and Use Committee of the University of Cincinnati. Male C57BL/6 db/db mice (Jackson Laboratories) were housed 4 per cage, exposed to a controlled 12 h/12 h light–dark cycle at room temperature (22 °C), and provided ab libitum access to water and a standard chow diet. Mice were randomized by ad libitum-fed blood glucose and body weight and were double-housed for the study. Test compounds were dissolved and administered as for DIO mice. Tail blood glucose levels were measured 0, 1, 3, 6, and 24 h after dosing, and every 24 h thereafter for 1 week.

## 3 Results

### 3.1 GLP-1/GIP/Gcg combination outperforms GLP-1/Gcg combination in weight reduction and glycemic control in DIO mice

**Figure 1:**
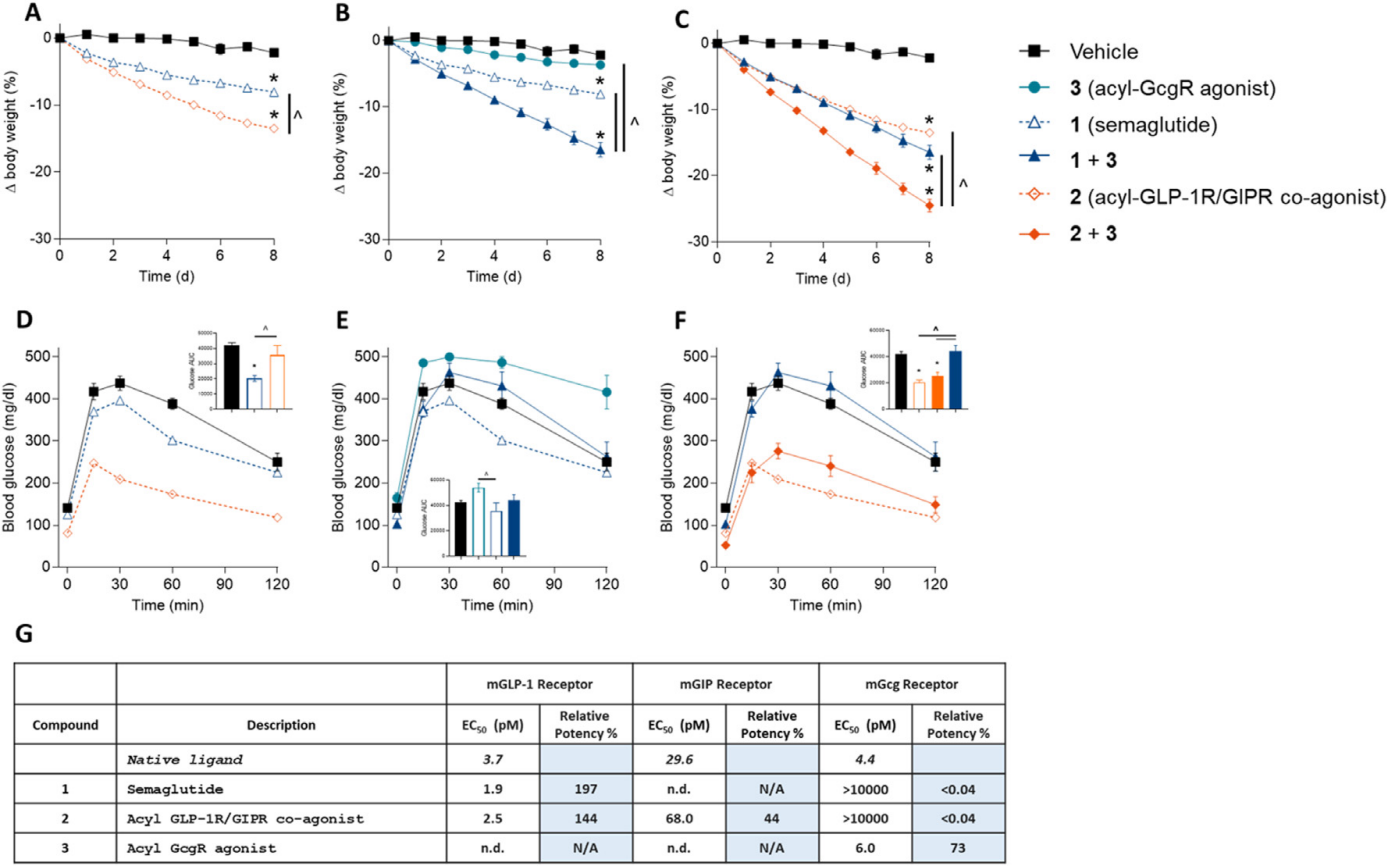
**Pharmacological effects of GLP-1R, GIPR, and GcgR agonism alone or in combination in DIO mice**. Body weight (A–C) and IPGTT blood glucose (D–F) for DIO mice. Animals were given subcutaneous injections once per day with semaglutide (**1**; 1 nmol/kg), acyl-GLP-1R/GIPR co-agonist (**2**; 1 nmol/kg), acyl-GcgR agonist (**3**; 3 nmol/kg), or combinations thereof. Body weight was measured daily over 8 days. The IPGTT was performed on day 8 (24 h after compound administration). *In vitro* potency at mouse-derived receptors is provided in panel G. Average starting body weight for mice in these studies was 62.6 g and did not differ significantly between any group. ∗ indicate a p-value < 0.05 compared to vehicle control; ˆ indicate a p-value < 0.05 relative to a treatment group as indicated.

To test the hypothesis that the combined effects of GLP-1, GIP, and Gcg on glycemic control and weight reduction are superior to that of the constituent factors, we performed a study comparing combinations of similarly acylated, long-acting agonists for GLP-1R (semaglutide, **1**) [47], GLP-1R/GIPR (**2**), and GcgR (**3**) in DIO mice (Figure 1). In vitro receptor activation assays indicated that **1** and **2** possessed similar potency at the mouse GLP-1R receptor with respect to cAMP production (Figure 1), permitting a straightforward examination of the role of the GIP and Gcg activities in mice. After eight days of once-daily treatment, **2** demonstrated robust improvements in body weight lowering and glycemic control compared to an equimolar dose of **1**, recapitulating previous data for shorter-acting molecules [34]. The combination of **1** and **3** similarly resulted in improved body weight lowering compared to either agent alone, though the glycemic benefit of semaglutide was eroded by the hyperglycemic activity of **3**. Finally, combination treatment with **2** and **3** resulted in both superior weight reduction and glycemic control over the **1**/**3** combination, highlighting the importance of the GIP component to contribute to further weight reduction in this context and also to provide additional buffering of the diabetogenic effect of GcgR agonism, even in the presence of super-stoichiometric amounts of **3**. Of note, combination treatment with **2** and **3** also resulted in superior weight reduction relative to **2** alone, albeit with a lessened improvement in glucose tolerance. With these promising results, we sought to develop a long-acting triple agonist bearing all three agonistic activities.

### 3.2 Optimization of in vitro receptor potency of triple agonists

Our design of long-acting GLP-1R/GIPR/GcgR triple agonists began upon the foundation of the clinical, once-daily GLP-1R/GIPR co-agonist NN0090-2746 (**4**, Table 1) [34]. We established two goals for our campaign: introduce GcgR potency into the molecule without sacrificing the GLP-1R and GIPR potency of **4**; and protract time-action through acylation with a fatty diacid, inspired by once-weekly acylated peptides such as semaglutide [47] and tirzepatide [35]. As previously described [34], a single Glu3Gln mutation (**5**) introduced GcgR agonism with little impact on incretin potency (Table 1). However, substitution of the palmitoylation at position 40 with the diacid-based acylation of semaglutide (OEG-OEG-γGlu-C18 diacid, as in compound **6**), rationally introduced to further extend time-action, resulted in loss of potency at all three receptors with respect to cAMP production, particularly GcgR. Importantly, this demonstrates that the chemical nature of the acylation influences not only the pharmacokinetics of the peptide but also the pharmacophore itself, which further highlights that such modifications are not inert bystanders with respect to biological activity [48] and biodistribution [49]. Therefore, we performed a scan of acylation positions throughout the peptide backbone to identify sites more accommodating of the diacid protractor, which identified position 16 (as in **7**) as a preferred site for this nature of acylation due to appreciable potency across all three receptors. The elimination half-life of **7** (Table 2), as measured after i.v. dose in minipigs, could be increased by nearly 40% through the replacement of the C18 diacid protractor with a C20 diacid (**8**), providing an even more attractive PK profile for potential once-weekly clinical administration when compared to the once-weekly GLP-1R agonist semaglutide [47].

**Table 1:** *In vitro* receptor activation of human-specific receptors by novel long-active triple agonists, compared to the native ligand for each receptor. **X** = 2-aminoisobutyric acid; **B** = K[OEG-OEG-γGlu-C18 diacid]; **J** = K[OEG-OEG-γGlu-C20 diacid]; **Z** = K[εLys-εLys-γGlu-C20 diacid].

Compound	Peptide Sequence	hGLP-1 Receptor		hGIP Receptor		hGcg Receptor	
		EC_50_ (pM)	Relative Potency %	EC_50_ (pM)	Relative Potency %	EC_50_ (pM)	Relative Potency %
	Native ligand	6.9		10.4		9.5	
4	YX**E**GT FTSDY SIYLD KQAAX EFVNW LLAGG PSSGA PPPS**K(C16)**—NH_2_	3.6	193	4.8	217	2333.8	0.4
5	YXQGT FTSDY SIYLD KQAAX EFVNW LLAGG PSSGA PPPS**K(C16)**—NH_2_	3.5	199	6.0	173	29.0	33
6	YXQGT FTSDY SIYLD KQAAX EFVNW LLAGG PSSGA PPPS**B**—NH_2_	11.7	59	44.5	23	703.4	1
7	YXQGT FTSDY SIYLD **B**QAAX EFVNW LLAGG PSSGA PPPS—NH_2_	7.4	93	27.4	38	81.9	12
8	YXQGT FTSDY SIYLD **J**QAAX EFVNW LLAGG PSSGA PPPS—NH_2_	6.8	101	26.7	39	163.4	6
9	YXQGT FTSDY SIYL**EJ**QAAX EFV**Q**W LL**E**GG PSSGA PPPS—NH_2_	3.1	220	11.4	92	185.0	5
10	YXQGT FTSDY SIYL**EZ**QAAX EFV**Q**W LL**E**GG PSSGA PPPS—NH_2_	2.8	244	7.0	150	86.6	11
11	YX**H**GT FTSDY SIYL**EZ**QAAX EFV**Q**W LL**E**GG PSSGA PPPS—NH_2_	106.4	7	7.1	147	72.1	13
12	**H**XQGT FTSDY SIYL**EZ**QAAX EFV**Q**W LL**E**GG PSSGA PPPS—NH_2_	1.7	410	55.0	19	357.1	3
13	**H**X**H**GT FTSDY SIYL**EZ**QAAX EFV**Q**W LL**E**GG PSSGA PPPS—NH_2_	2.0	340	7.1	146	48.7	19
14	**H**X**H**GT FTSDY SIYL**EZ**KAAX EFV**Q**W LL**E**GG PSSGA PPPS—NH_2_	3.3	211	7.3	142	13.7	69
15	**H**X**H**GT FTSDY SIYL**EZ**Q**Y**AX EFV**Q**W LL**E**GG PSSGA PPPS—NH_2_	6.8	101	7.7	135	13.1	72
16	**H**X**H**GT FTSDY SIYL**EZKY**AX EFV**Q**W LL**E**GG PSSGA PPPS—NH_2_	3.9	177	15.6	67	9.3	102

**Table 2:** . Pharmacokinetic parameters after a single intravenous dose of 2 nmol/kg in minipigs (n = 2–3). Data for semaglutide is taken from Lau et al. [47]].

	Cl (L/h/kg)	Vz (L/kg)	T_1/2_ (h)
Semaglutide, 1	0.0016	0.102	46
7	0.0014	0.121	61
8	0.0012	0.154	85
16	0.0015	0.164	75

Because the potency of these diacid-protracted molecules was still reduced at all receptors compared to parent **5**, our attention focused on improving overall potency through select modifications of the peptide backbone and linker region (Table 1). A significant improvement in cAMP potency was observed with a dual mutation of position 1 and position 3 to histidine (**13**), even though each individual mutation alone (**11**, **12**) hindered potency at one or more receptors. As we sought to test the effect of more aggressive GcgR potency on both weight loss and glycemic control, we explored additional mutations to increase the GcgR potency of **13** with a more balanced ratio with respect to GLP-1R potency. A Q17K mutation (**14**) and a A18Y mutation (**15**) improved GcgR potency with slight to moderate reduction in GLP-1R potency, and a combination of these mutations (**16**) resulted in a compound with potencies at both GLP-1R and GcgR comparable to the native ligands.

Ultimately, this mutational “fine-tuning” provided a suite of high-potency triple agonists with subtle variations in receptor activity ratios that could be used to interrogate optimal activity balance. However, differences in *in vitro* cAMP potencies between human receptors and those of different pre-clinical models, particularly with regard to GIPR, can complicate translation of pre-clinical results to a clinical setting [44]. Therefore, triple agonists were assayed at mouse-derived GLP-1R, GIPR, and GcgR (mGLP-1R, mGIPR, and mGcgR, respectively) along with comparator compounds to investigate *in vitro* cAMP potencies and subsequently support the appropriate in vivo pharmacodynamic comparisons in DIO mice. The presence of Lys at position 17 (as in **16**) was found to be beneficial for mGIPR potency despite having a modestly detrimental effect for hGIPR potency. The high potencies and relative balance of **16** at all three mouse-specific receptors, as well as the long half-life of this compound in minipigs (75 h, Table 2) encouraged us to take this compound forward for pharmacodynamic evaluation in DIO mice.

### 3.3 Metabolic effects of triple agonists in DIO mice

**Figure 2:**
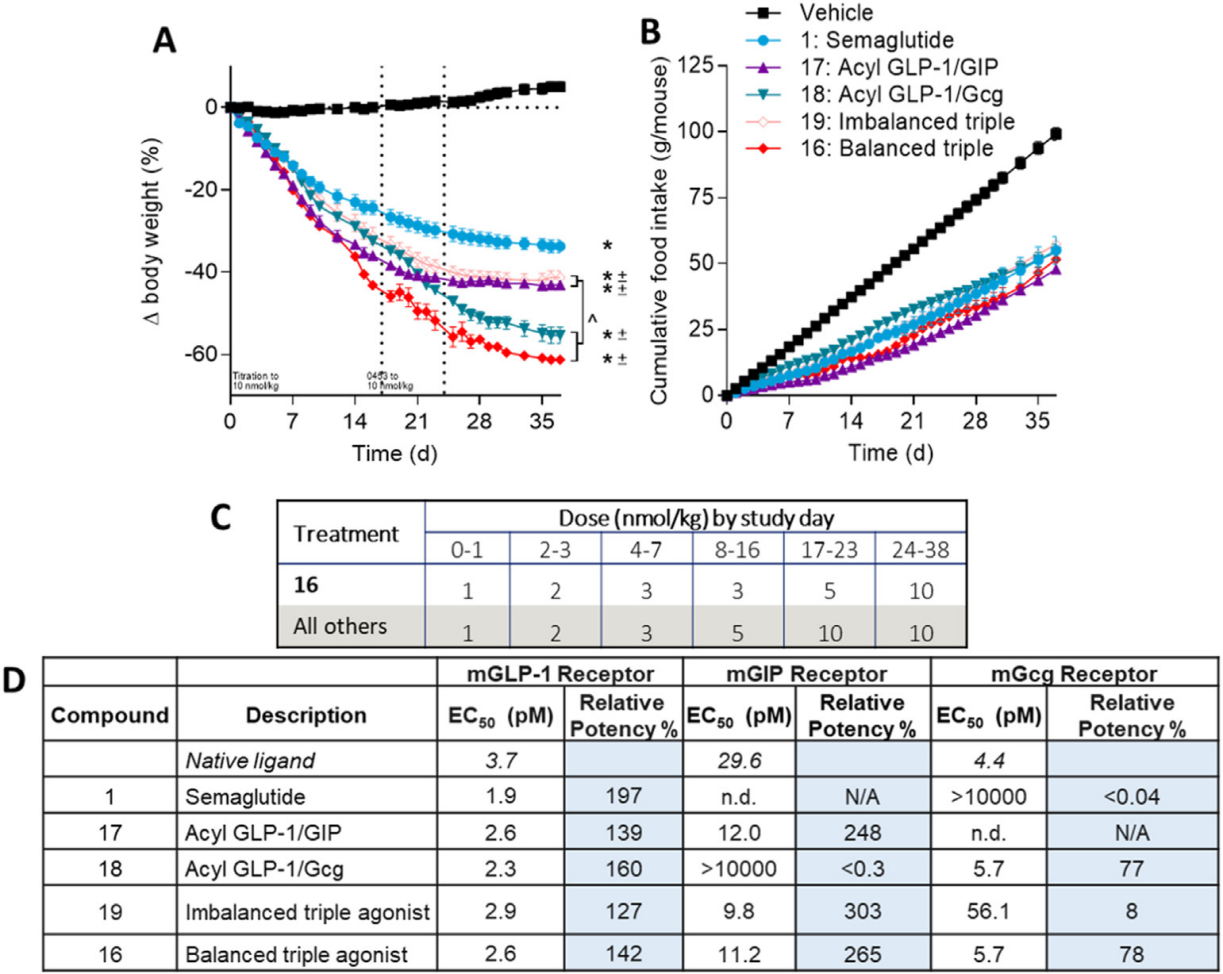
**GcgR agonism provides additional body weight lowering efficacy over GLP-1R agonism and GLP-1R/GIPR co-agonism in DIO mice**. Body weight (A) and food intake (B) for DIO mice given subcutaneous injections once per day with semaglutide **1**, acyl-GLP-1R/GIPR co-agonist **17**, acyl-GLP-1R/GcgR co-agonist **18**, imbalanced GLP-1R/GIPR/GcgR triple agonist **19**, and balanced GLP-1R/GIPR/GcgR triple agonist **16**. Dosing concentrations and dose-escalation schedule is provided in panel C. The *in vitro* potency at mouse-derived receptors is provided in panel D. Average starting body weight for mice in these studies was 59.0 g and did not differ significantly between any group. ∗ indicate a p-value < 0.05 compared to vehicle control; ˆ indicate a p-value < 0.05 relative to a treatment group as indicated.

Compound **16** dose-dependently reduced body weight and food intake in DIO mice while maintaining improvements in glycemic control compared to vehicle (Supplemental Fig. 1). Body weight reductions greater than 30% were achieved after 2 weeks of treatment with a 3 nmol/kg daily dose. We next sought to compare the maximal weight lowering efficacy of the balanced triple agonist **16** to similarly acylated comparators possessing GLP-1R agonism alone (semaglutide), GLP-1R/GIPR co-agonism (**17**), GLP-1R/GcgR co-agonism (**18**), or to an “imbalanced” triple agonist (**19**) with 10-fold lower mGcgR potency compared to **16** (Figure 2). All compounds possessed comparable *in vitro* potency at mGLP-1R, which allowed for a delineation of the contributions of the GIP and Gcg activities. To ensure tolerability, doses of all compounds were up-titrated from 1 nmol/kg to 10 nmol/kg over 24 days, then maintained at the 10 nmol/kg dose for up to 18 days. Animals that achieved the average weight of lean litter mates (22.5 g) were removed from the study as this criterion was determined to be normalization of body weight; the number of animals achieving normalized body weight and day at which they achieved normalization of body weight are detailed in Supplemental Table 1. Mean body weight loss was calculated from all animals remaining in the study on a given day. Five of the eight animals treated with **16** achieved body weight normalization, accounting for an unprecedented 55–60% body weight loss. Differentiation of **16** from all comparators was also apparent within the first two weeks of treatment, providing evidence for the contributions of all three constitutive activities to enhance weight lowering potency in addition to maximal efficacy. Furthermore, the influence of GcgR agonism to influence maximal weight lowering efficacy was readily apparent with the recently-reported clinical GLP-1/Gcg co-agonist **18** [50], as well as a sharp differentiation in performance between balanced triple agonist **16** and imbalanced triple agonist **19**. Despite the wide range of weight lowering efficacy, food intake suppression was similar among all treatment groups, further highlighting the impact of harnessing distinct mechanisms of body weight reduction, independent of food intake, that complement the GLP-1R mediated effects on food intake. These findings were also largely recapitulated in DIO rats treated with balanced triagonist **20** (Supplemental Fig. 4).

**Figure 3:**
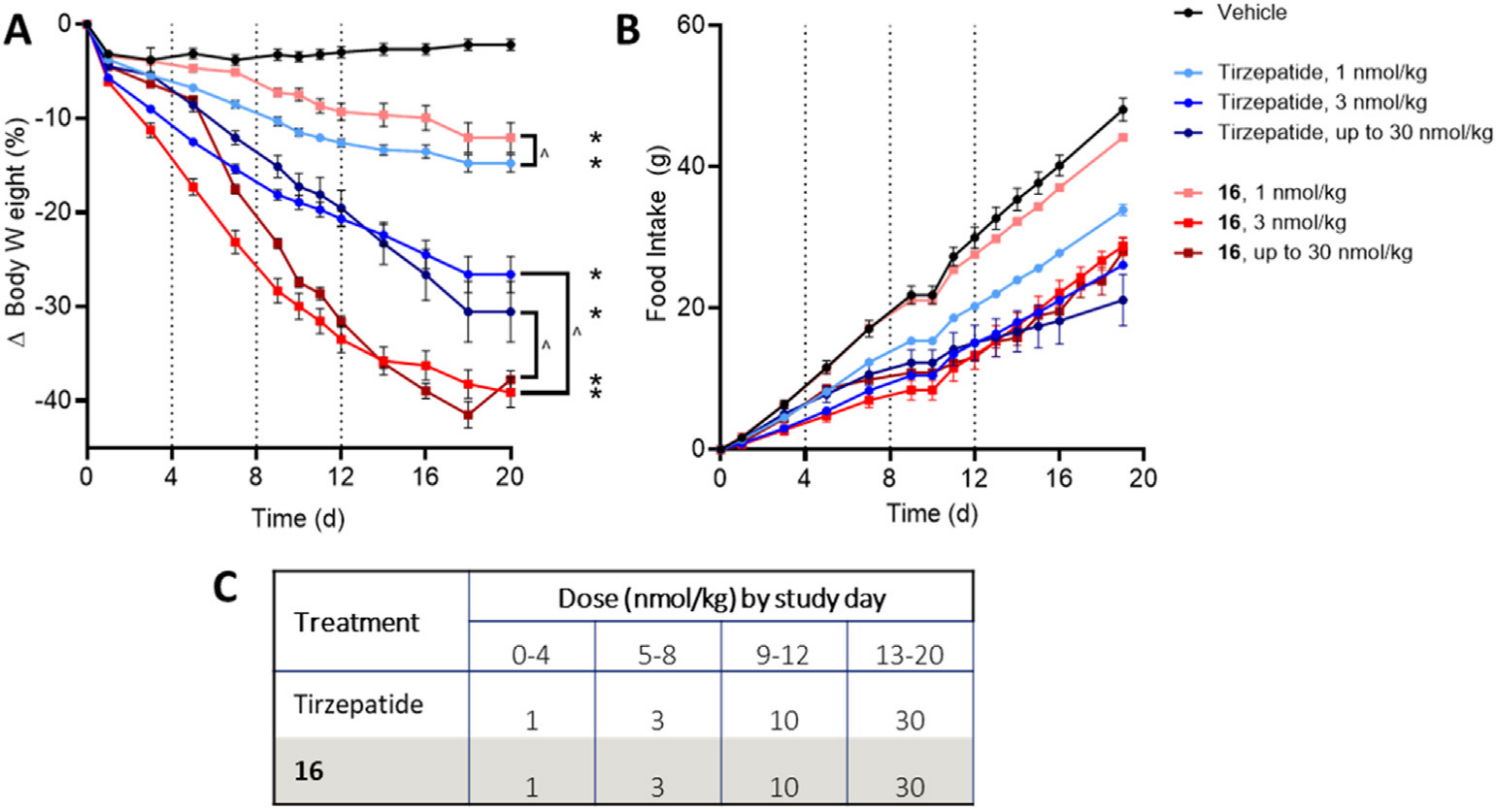
**Triple receptor agonism of GLP-1R, GIPR, and GcgR produces superior body weight lowering efficacy compared to GLP-1/GIPR co-agonist tirzpatide in DIO mice**. Body weight (A) and food intake (B) for DIO mice given subcutaneous injections once per day with tirzepatide or GLP-1R/GIPR/GcgR triple agonist **16**. Dosing concentrations and dose-escalation schedule is provided in panel C. Average starting body weight for mice in these studies was 61.2 g and did not differ significantly between any group. ∗ indicate a p-value < 0.05 compared to vehicle control; ˆ indicate a p-value < 0.05 relative to the equimolar dose of tirzepatide as indicated.

Next, we compared our triple agonist **16** against GLP-1/GIP co-agonist tirzepatide, the most clinically advanced long-acting, incretin-based mixed agonist that shows clear differentiation from GLP-1R mono-agonists in preclinical studies as well as in clinical studies [35,41]. DIO mice were treated with daily doses of 1 nmol/kg, 3 nmol/kg, or an escalation from 1 nmol/kg to 30 nmol/kg of either compound (Figure 3). Both compounds demonstrated a strong correlation between dose, body weight lowering, and food intake suppression, with **16** showing a steeper dose response reflective of the glucagon constituent. While treatment with either **16** or tirzepatide at a dose of 1 nmol/kg resulted in similar weight loss after 20 days of treatment, both the 3 nmol/kg and dose-escalated groups demonstrated significantly more weight lowering efficacy with **16** treatment than tirzepatide, despite comparable food intake suppression in all four groups. This further supports the notion that a threshold level of GcgR agonism is required for the full impact of triple agonism on body weight lowering. Importantly, groups treated with the higher doses of **16** showed similar improvements in glycemic control relative to groups treated with tirzepatide (Supplemental Fig. 3). This provides further evidence that dual incretin activity can not only offset the hyperglycemic effects of glucagon activity, but even overcome it to yield net improvements in glycemic control comparable to a dual incretin alone.

**Figure 4:**
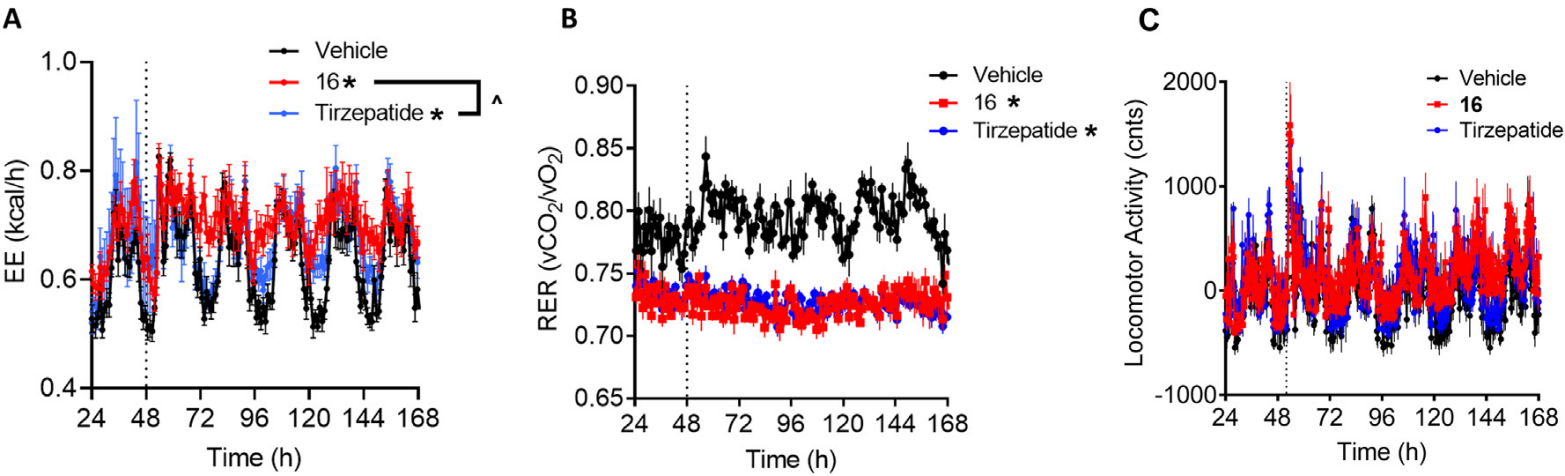
**Triple receptor agonism of GLP-1R, GIPR, and GcgR but not the dual receptor agonism of GLP-1 and GIPR induces energy expenditure in DIO mice**. Energy expenditure (A), respiratory exchange ratio (B) and locomoter activity (C) for DIO mice given subcutaneous injections once per day with tirzepatide (3 nmol/kg) or GLP-1R/GIPR/GcgR triple agonist **16** (3 nmol/kg). ∗ indicate a p-value < 0.05 compared to vehicle control; ˆ indicate a p-value < 0.05 relative to tirzepatide as indicated.

To unequivocally demonstrate the contribution of glucagon-stimulated increased energy expenditure to body weight reduction, DIO mice were treated with daily injections of 3 nmol/kg of **16** or tirzepatide for 5 days inside an indirect calorimeter (Figure 4). Treatment with **16** resulted in increased energy expenditure over both vehicle and tirzepatide, confirming the expected contribution of glucagon receptor agonism to weight lowering efficacy in this triple agonist independent of differences in food intake [43]. The energy expenditure increase was not accompanied by a change in locomotor activity (Figure 4C) as demonstrated previously with triagonists [43].

**Figure 5:**
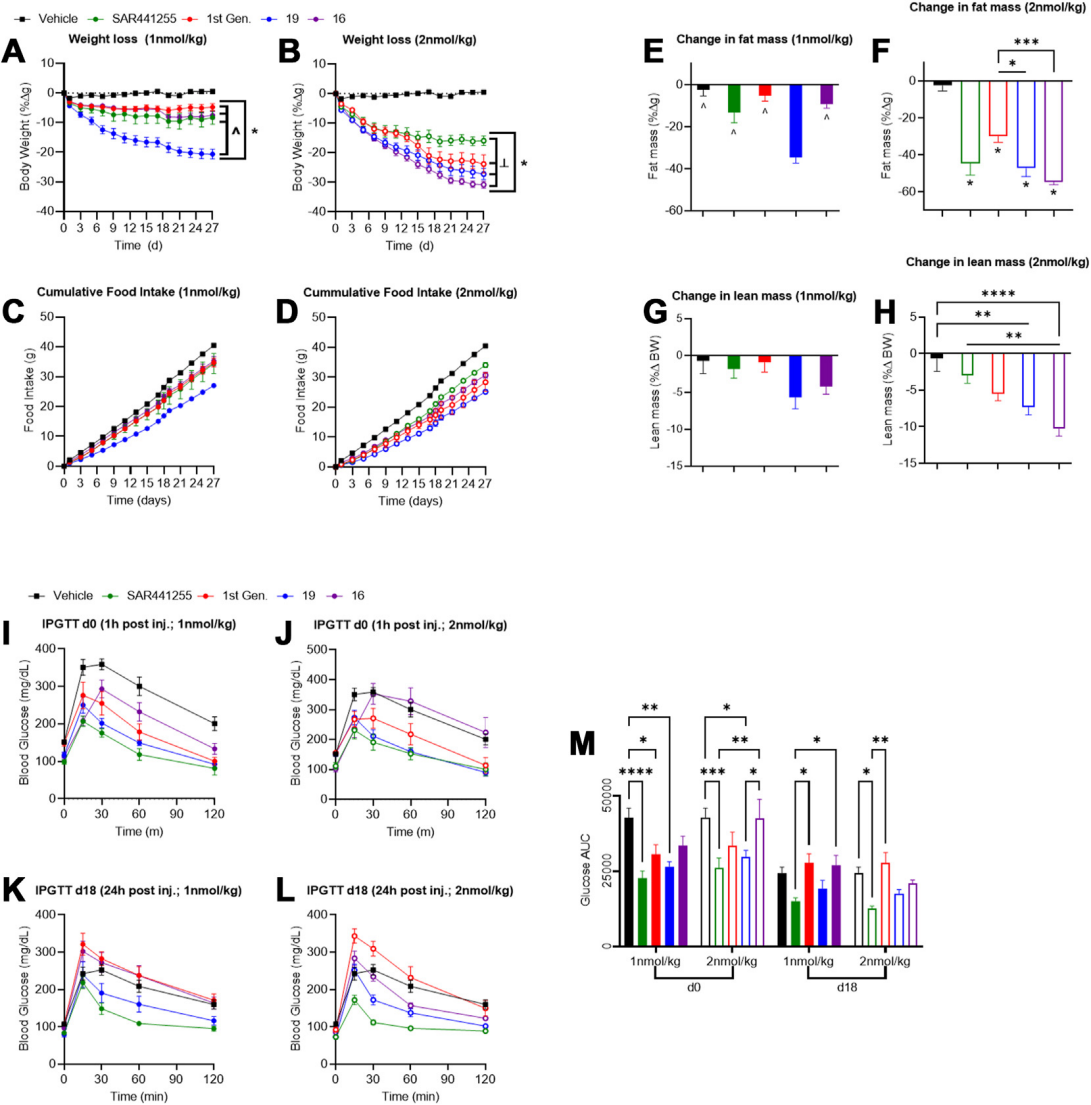
**Comparison of in vivo efficacy of next-generation triagonists to published triagonists**. Weight-loss (A,B), food intake (C,D) fat mass (E,F), and lean mass (G,H) for DIO mice treated with either vehicle (black), SAR441255 (green), first generation triagonist (red), **19** (blue), or **16** (purple) at either 1 nmol/kg (A,C,E,G; solid symbols) or 2 nmol/kg (B,D,F,H; open symbols). Glucose excursion during an IPGTT in DIO mice treated with indicated compounds either 1 h after injection on day 0 of the studies (I,J) or 24 h after compound injection on day 18 (K,L) and glucose area under the curve (AUC; M). Average starting body weight for mice in these studies was 60.2 g and did not differ significantly between any group. ∗ indicate p-value > 0.05 compared to vehicle or as indicated; ˆ indicate p-value > 0.05 between groups as indicated; ╧ indicate p-value > 0.05 compared to SAR441255.

Finally, the efficacy of these next-generation balanced (**16**) and imbalanced (**19**) triagonists for weight reduction, food intake, and glucose control was further benchmarked against the published first generation triagonist [43] as well as the recently disclosed, balanced triagonist SAR441255 [51]. Mice treated with high dose (2 nmol/kg) **16** or **19** showed significantly superior body weight loss compared to both the first generation triagonist and SAR441255 (Figure 5B). Conversely, **19** showed increased weight loss compared to all compounds tested at lower doses (1 nmol/kg, Figure 5A); the dose response curve for **16** was notably steeper than all other compounds in keeping with the dose titration response observed in Figure 2A. The weight loss observed in mice treated with **16**, **19**, and the first generation triagonist was predominantly derived from a reduction in fat mass, not lean mass (Figure 5E–H). However, there was no significant difference in fat mass reduction but significant changes in lean mass reduction between SAR441255 and **16**, indicating lean mass constitutes a meaningful amount of the overall weight loss difference induced by these compounds. Glucose control was improved by all compounds when administered at low doses 1 h prior to an IPGTT (Figure 5I,J,M). The degree of improvement is largely predicated by glucagon receptor potency with SAR441255 and **19** providing the greatest degree of improvement. Glucose control was improved in mice treated with **19**, SAR441255, and the first generation triagonist but not **16** in an IPGTT performed after chronic dosing, 24 h following final injection (Figure 5K,L,M). Plasma triglycerides, ALT, AST were unchanged in any group, whereas circulating cholesterol was decreased at both doses in all groups consistent with previous studies (Supplemental Fig. 5) [43]. Leptin levels were measured as a broad marker of adiposity. Leptin was decreased in all groups at the higher dose compared to vehicle (Supplemental Fig. 5), whereas insulin levels, an indicator of insulin sensitivity, were only decreased in the first generation triagonist and **16** and **19** treated groups at the higher dose (Supplemental Fig. 5).

## 4 Discussion

Obesity prevalence is escalating due in part to, but not limited to, the minimal efficacy of lifestyle interventions and the inaccessibility of effective yet invasive treatments like bariatric surgery [52]. This makes pharmacologic intervention for weight loss an increasingly critical modality for patient care. GLP-1R agonists can effectively lower body weight in obese patients to levels not achieved before with anti-obesity therapies [11,53]; however, weight loss seems to plateau over time and with diminishing gains as dose is increased within tolerable ranges [11]. Thus, there is a clear need for agents that enhance weight loss above that of GLP-1R agonists alone. Here, we demonstrate a superior weight-lowering effect of rationally-designed, next-generation single molecule GLP-1/GIP/Gcg triagonists relative to semaglutide, tirzepatide, other chemically/pharmacokinetically matched dual agonists, and pharmacologically disparate triagonists in DIO mice. Furthermore, the optimal triagonist safely normalized body weight in these mice with chronic treatment, a phenomenon that has not been shown before with pharmacotherapy alone. These data also show the addition and potency of GcgR agonism as a determinate factor in the weigh-lowering and weight-normalizing efficacy of this triple receptor agonism, and the inclusion of GIPR agonism not only contributes to the weight-lowering efficacy but permits maximal GcgR engagement without a deterioration in glucose control.

Previous studies in preclinical models demonstrate the potential for GcgR agonism to induce weight loss either alone [54,55] or in combination with GLP-1R and/or GIPR agonism [43]. However, this multi-receptor agonism approach to weight loss was hindered by commercial considerations of production and formulation [45], the biological impasse that glucagon potently induces hyperglycemia [56,57], and potential dose-limiting cardiovascular liabilities arising from positive chronotropic actions to influence hemodynamic parameters [58]. The invention of monomeric triple receptor agonists capable of activating the GLP-1R, GIPR, and GcgR provides an apparent resolution to these issues. These compounds, termed triagonists or triple agonists, couple the weight-reducing efficacy of GLP-1R, GIPR, and GcgR agonism while simultaneously buffering the glycemic effects of Gcg with dual incretin receptor activation [43,45]. The resultant compounds deliver effective glycemic control and weight reduction in preclinical studies [43,59] with anticipated efficacy in emergent clinical studies. However, the individual constituent mechanisms of action that contribute to the observed weight loss are incompletely understood. The data presented here demonstrate that the potency of GcgR agonism defines the maximal weigh-lowering capacity of the triple agonist, presumably through multiple mechanisms of increased energy expenditure, insofar as increasing *in vitro* GcgR potency with respect to cAMP production prognosticates *in vivo* weight reduction. Furthermore, our data demonstrate that a triple agonist with balanced activity at the three receptors produces greater maximal weight loss compared to tirzepatide, a dual GLP-1R and GIPR agonist that shows remarkable clinical efficacy. Critically, our data suggest that the differentiating factor for this superior weight loss effect is achieved through an increase in energy expenditure, given the elevated energy expenditure in combination with similar reductions in food intake between maximally effective triple agonist and tirzepatide (and other co-agonist) groups. This is consistent with the known role of GLP-1R [49,60] and GIPR [37,38] agonism to reduce food intake, primarily but not exclusively via CNS mediated signaling events, and GcgR agonism to induce energy expenditure [54] through mechanisms that are not fully described. It has been reported in both clinical and preclinical settings that GIPR agonism helps to ameliorate the adverse nauseating effects of GLP-1R agonists [[39], [42]]. While the appropriate assay systems to test this outcome are not in place for these preclinical studies in mice, it is an interesting possibility that triagonists might be able to induce greater weight-loss without additional adverse events. Although speculative, these mechanisms likely include canonical and non-canonical thermogenesis as well as macronutrient futile cycling, notably glucose when paired with incretin receptor agonism.

The superiority of triple agonists over dual incretin receptor agonists like tirzepatide in preclinical models suggests the inclusion and relative potency of GcgR agonism as the primary determinant of weight-reducing efficacy for these compounds. This is further demonstrated by the superiority of a second-generation triagonist with heighted potency at the GcgR for weight loss compared to less potent, first-generation triagonists. Optimization of GcgR agonism for weight loss is in its infancy; however, insight into this process may be gleaned from recent characterization of dual incretin receptor agonists. First, largely speculative arguments have been mounted regarding the optimal potency ratio between two or more receptors. However, *in vitro* potency and preclinical efficacy data directly assessing these relationships is lacking and confounded by the apparent reduction in potency of GIPR agonists in rodents [61] and the compensatory sensitization of incretin receptors in genetic knockout mouse lines [62,63]. Thus, adequately assessing how receptor potency ratio affects in vivo efficacy in the context of GIPR agonism requires extensive and costly clinical investigation. Secondly and similarly, there is ongoing debate as to whether tirzepatide activation of the GIPR contributes to its efficacy as suggested by the clinical and preclinical data [61]. While GIPR antagonism has been shown to reduce adiposity in preclinical models [64], the clinical data show tirzepatide, a dual GLP-1R/GIPR agonist, produces greater weight loss and glucose lowering than the GLP-1R agonist semaglutide alone [42], particularly at doses which comparably engage the GLP-1R [61]. This suggests that the addition of GIPR agonism is responsible for meaningful differences in clinical outcomes. Tests in human subjects to ascertain the relative contribution of each constitutive receptor activity to the pharmacology of triple agonists are difficult even with emerging tracers to estimate receptor occupancy in vivo, but a robust analysis of emerging clinical trials may allow for a crude deduction of this information. Third, tirzepatide has been demonstrated to be a biased GLP-1R agonist exhibiting largely retained efficacy with respect to cAMP production, albeit with reduced potency, but significantly reduced β-arrestin recruitment [61]. This biased signaling profile is known to contribute to the insulin secretory capacity of tirzepatide in isolated cell systems and is broadly thought to improve GLP-1R agonist efficacy in preclinical studies, but any therapeutic benefit over unbiased signaling in clinical settings remains to be proven. While cursory efforts have been devoted to characterizing the structure activity relationship responsible for this signaling profile at the GLP-1R [65], the structural and sequence determinants of biased agonism at GIPR and GcgR are largely unknown, and thus would add another level of complexity to the structure–activity relationships leveraged in the rational design of these unimolecular triple agonists.

The pharmacologic mechanisms by which triple agonism of the GLP-1R, GcgR, and GIPR influences weight loss are worth considering in the context of optimization of receptor engagement. GLP-1R agonists are thought to primarily mediate weight loss by reducing food intake [49,53]. There is observational data to infer an additive and possibly synergistic effect of exercise with GLP-1R agonism on weight loss, suggesting non-anorectic mechanisms that are not well characterized [5,66]. The contribution of GcgR agonism to weight loss appears to be primarily mediated by an increase in energy expenditure as suggested by our data; it is hypothesized that hepatic glucose mobilization for consumption in peripheral tissues, including the fat, is primarily responsible. The weight loss contribution of GIPR agonism is less clear. Initial reports that GIPR null mice [64] are protected from diet-induced obesity have been supported by preclinical studies in rodents and non-human primates that GIPR antagonism induces additive weight loss when combined with GLP-1R agonism [32,67], and human genetics data that loss-of-function GIPR variants associate with reduced adiposity [68]. However, recent data provides human genetic evidence that supports a beneficial role of sustained GIP signaling on cardiometabolic health outcomes, notably a lower BMI [69]. Further, preclinical demonstrations that GIPR agonism induces significant weight loss through CNS GIPR activation [38] align with emerging clinical data of tirzepatide, particularly as compared head-to-head against GLP-1R agonist semaglutide [42], to suggest that activation of GIPR is advantageous for weight loss. How GIPR antagonism and agonism regulate body weight is unclear, but it has been suggested that there are common mechanisms of antagonism and agonism to drive desensitization of GIPR, although the same effect has been observed with GLP-1R manipulation [70]. Recent evidence suggests that GIPR activity may modulate GLP-1 dependent feeding behavior by reducing the emetic activity promoted that GLP-1R in the hindbrain [39], an area of the brain relatively exposed to small circulating peptides. Thus, it is plausible that differential biodistribution of the pharmacologic compound or chemical modality used (i.e. peripherally restricted antibody vs. brain-permeable peptide by nature of the fatty acid used **49**) may lead to the cell or tissue-selective engagement of their targeted receptors.

Weighing the therapeutic potential of triple agonists for diabetes and obesity against the cardiovascular effects of GcgR agonism specifically is a necessary consideration. Glucagon is broadly thought to have cardio-stimulatory effects via GcgR expressed on the myocardium [58]. GcgR mono-agonists are known to increase heart rate and contractility, which has led to the suggestion for them to be used as a treatment for low cardiovascular output in acute settings. However, human efficacy data for this strategy is scant. Recent reports demonstrate that triple agonists exert a transient stimulatory effect on heart rate [71], akin to that observed with preclinical studies of GLP-1R agonists. This finding is notable given the demonstration from large scale cardiovascular outcomes trials that GLP-1R agonism is largely cardioprotective [72], and preclinical data that tachycardia observed with GLP-1R agonists dissipates over time or is minimized in the clinic with dose escalation [73,74]. Thus, it possible that dose escalation regimens, which are standard of care for available GLP-1R agonists to minimize gastrointestinal adverse events, may mitigate these apparently detrimental outcomes of GcgR agonists on heart rate but must be empirically proven in a clinical setting.

## 5 Conclusions

Previous studies have demonstrated that pharmacologic activation of GLP-1R, GIPR, and GcgR provides significant body weight loss in preclinical models, and emerging clinical data of compounds that engage two of these receptors are showing the translation of these preclinical results into human subjects. While GLP-1R and GIPR activation are necessary to buffer the hyperglycemic effects of glucagon pharmacology, we demonstrate herein that a long-acting, balanced triple receptor agonist reduces body weight in a manner superior to that achieved by mono- or dual-incretin receptor agonism alone, notably outperforming the clinical assets semaglutide and tirzepatide, and a related clinical asset SAR441255. It should be noted that compound **16** induces a notable reduction in lean mass and glucose control compared to both **18** and SAR44125. This phenomenon appears to be positively correlated to *in vitro* GcgR potency, indicating a potential contribution of GcgR mediated amino acid catabolism and muscle wasting to the weight-loss phenomenon of **16** in this preclinical study [75], while elevated glycogenolysis and gluconeogenesis may contribute to the glucose control outcome [75]. Optimization of the peptide backbone and acylation resulted in triple agonists with in vitro potency equaling or surpassing that of the native hormone for each receptor and pharmacokinetic properties supporting once-weekly clinical administration. Characterization in DIO mice revealed unprecedented efficacy to lower body weight to the point of weight normalization and reversal of obesity. This weight loss is proportional to the degree of GcgR engagement, at least as assessed *in vitro*, and functionally mediated by an increase in energy expenditure. These data provide clear evidence for the potency of glucagon pharmacology to tune the maximal weight loss that can be achieved pre-clinically, and eventual clinical testing will provide the translational evidence of the power in harnessing these three pharmacologies for patients with obesity. Additionally, these data also suggest a series of considerations for the future discovery, development, and optimization of triple receptor agonists on this nature.

## Author contributions

P.J.K., S.A.M, J.D.D, and B.F. wrote the manuscript. P.J.K., B·P., and Y.H. designed, synthesized, and characterized compounds. S.A.M. and A.M.K·H performed in vitro experiments. J.D.D., K.R.H., A.K.O. and D.P.-T. designed in vivo experiments. K.R.H. and D.P.-T. supervised in vivo experiments. R.D.D. and B.F. conceptualized research strategy and designed experiments. All authors interpreted data.

## References

[1] Williams D.M., Nawaz A., Evans M. (2020). Drug therapy in obesity: a review of current and emerging treatments. Diabetes Therapy.

[2] Müller T.D., Clemmensen C., Finan B., DiMarchi R.D., Tschöp M.H. (2018). Anti-obesity therapy: from rainbow pills to polyagonists. Pharmacological Reviews.

[3] Jones B.J., Bloom S.R. (2015). The new era of drug therapy for obesity: the evidence and the expectations. Drugs.

[4] Mehta A., Marso S.P., Neeland I.J. (2017). Liraglutide for weight management: a critical review of the evidence. Obesity Science and Practice.

[5] Wilding J.P.H., Batterham R.L., Calanna S., Davies M., Van Gaal L.F., Lingvay I. (2021). Once-weekly semaglutide in adults with overweight or obesity. New England Journal of Medicine.

[6] Wadden T.A., Bailey T.S., Billings L.K., Davies M., Frías J.P., Koroleva A. (2021). Effect of subcutaneous semaglutide vs placebo as an adjunct to intensive behavioral therapy on body weight in adults with overweight or obesity: the STEP 3 randomized clinical trial. JAMA.

[7] Rubino D., Abrahamsson N., Davies M., Hesse D., Greenway F.L., Jensen C. (2021). Effect of continued weekly subcutaneous semaglutide vs placebo on weight loss maintenance in adults with overweight or obesity: the STEP 4 randomized clinical trial. JAMA.

[8] O'Neil P.M., Birkenfeld A.L., McGowan B., Mosenzon O., Pedersen S.D., Wharton S. (2018). Efficacy and safety of semaglutide compared with liraglutide and placebo for weight loss in patients with obesity: a randomised, double-blind, placebo and active controlled, dose-ranging, phase 2 trial. Lancet.

[9] Bettge K., Kahle M., Abd El Aziz M.S., Meier J.J., Nauck M.A. (2017). Occurrence of nausea, vomiting and diarrhoea reported as adverse events in clinical trials studying glucagon-like peptide-1 receptor agonists: a systematic analysis of published clinical trials. Diabetes, Obesity and Metabolism.

[10] Raccah D. (2017). Safety and tolerability of glucagon-like peptide-1 receptor agonists: unresolved and emerging issues. Expert Opinion on Drug Safety.

[11] Frías J.P., Auerbach P., Bajaj H.S., Fukushima Y., Lingvay I., Macura S. (2021). Efficacy and safety of once-weekly semaglutide 2.0 mg versus 1.0 mg in patients with type 2 diabetes (SUSTAIN FORTE): a double-blind, randomised, phase 3B trial. Lancet Diabetes & Endocrinology.

[12] Petersen J., Strømgaard K., Frølund B., Clemmensen C. (2019). Designing poly-agonists for treatment of metabolic diseases: challenges and opportunities. Drugs.

[13] Anamika S., Ved S. (2018). Multi-functional chimeric peptides: the more the merrier. Protein and Peptide Letters.

[14] Camilleri M., Acosta A. (2018). Combination therapies for obesity. Metabolic Syndrome and Related Disorders.

[15] Knerr P.J., Finan B., Gelfanov V., Perez-Tilve D., Tschöp M.H., DiMarchi R.D. (2018). Optimization of peptide-based polyagonists for treatment of diabetes and obesity. Bioorganic & Medicinal Chemistry.

[16] Tschöp Matthias H., Finan B., Clemmensen C., Gelfanov V., Perez-Tilve D., Müller Timo D. (2016). Unimolecular polypharmacy for treatment of diabetes and obesity. Cell Metabolism.

[17] González-García I., Milbank E., Diéguez C., López M., Glucagon Contreras C. (2019). GLP-1 and thermogenesis. International Journal of Molecular Sciences.

[18] Kleinert M., Sachs S., Habegger K.M., Hofmann S.M., Müller T.D. (2019). Glucagon regulation of energy expenditure. International Journal of Molecular Sciences.

[19] Salem V., Izzi-Engbeaya C., Coello C., Thomas D.B., Chambers E.S., Comninos A.N. (2016). Glucagon increases energy expenditure independently of brown adipose tissue activation in humans. Diabetes, Obesity and Metabolism.

[20] Sánchez-Garrido M.A., Brandt S.J., Clemmensen C., Müller T.D., DiMarchi R.D., Tschöp M.H. (2017). GLP-1/glucagon receptor co-agonism for treatment of obesity. Diabetologia.

[21] Nahra R., Wang T., Gadde K.M., Oscarsson J., Stumvoll M., Jermutus L. (2021). Effects of cotadutide on metabolic and hepatic parameters in adults with overweight or obesity and type 2 diabetes: a 54-week randomized phase 2b study. Diabetes Care.

[22] Parker V.E.R., Robertson D., Wang T., Hornigold D.C., Petrone M., Cooper A.T. (2019). Efficacy, safety, and mechanistic insights of cotadutide, a dual receptor glucagon-like peptide-1 and glucagon agonist. The Journal of Cinical Endocrinology and Metabolism.

[23] Ambery P., Parker V.E., Stumvoll M., Posch M.G., Heise T., Plum-Moerschel L. (2018). MEDI0382, a GLP-1 and glucagon receptor dual agonist, in obese or overweight patients with type 2 diabetes: a randomised, controlled, double-blind, ascending dose and phase 2a study. Lancet.

[24] Tillner J., Posch M.G., Wagner F., Teichert L., Hijazi Y., Einig C. (2019). A novel dual glucagon-like peptide and glucagon receptor agonist SAR425899: results of randomized, placebo-controlled first-in-human and first-in-patient trials. Diabetes, Obesity and Metabolism.

[25] Visentin R., Schiavon M., Göbel B., Riz M., Cobelli C., Klabunde T. (2020). Dual glucagon-like peptide-1 receptor/glucagon receptor agonist SAR425899 improves beta-cell function in type 2 diabetes. Diabetes, Obesity and Metabolism.

[26] Eriksson O., Haack T., Hijazi Y., Teichert L., Tavernier V., Laitinen I. (2020). Receptor occupancy of dual glucagon-like peptide 1/glucagon receptor agonist SAR425899 in individuals with type 2 diabetes. Scientific Reports.

[27] Henderson S.J., Konkar A., Hornigold D.C., Trevaskis J.L., Jackson R., Fritsch Fredin M. (2016). Robust anti-obesity and metabolic effects of a dual GLP-1/glucagon receptor peptide agonist in rodents and non-human primates. Diabetes, Obesity and Metabolism.

[28] Day J.W., Gelfanov V., Smiley D., Carrington P.E., Eiermann G., Chicchi G. (2012). Optimization of co-agonism at GLP-1 and glucagon receptors to safely maximize weight reduction in DIO-rodents. Peptide Science.

[29] Finan B., Müller T.D., Clemmensen C., Perez-Tilve D., DiMarchi R.D., Tschöp M.H. (2016). Reappraisal of GIP pharmacology for metabolic diseases. Trends in Molecular Medicine.

[30] Samms R.J., Coghlan M.P., Sloop K.W. (2020). How may GIP enhance the therapeutic efficacy of GLP-1?. Trends in Endocrinology and Metabolism.

[31] Holst J.J., Rosenkilde M.M. (2020). GIP as a therapeutic target in diabetes and obesity: insight from incretin Co-agonists. The Journal of Cinical Endocrinology and Metabolism.

[32] Killion E.A., Chen M., Falsey J.R., Sivits G., Hager T., Atangan L. (2020). Chronic glucose-dependent insulinotropic polypeptide receptor (GIPR) agonism desensitizes adipocyte GIPR activity mimicking functional GIPR antagonism. Nature Communications.

[33] Killion E.A., Wang J., Yie J., Shi S.D.-H., Bates D., Min X. (2018). Anti-obesity effects of GIPR antagonists alone and in combination with GLP-1R agonists in preclinical models. Science Translational Medicine.

[34] Finan B., Ma T., Ottaway N., Müller T.D., Habegger K.M., Heppner K.M. (2013). Unimolecular dual incretins maximize metabolic benefits in rodents, monkeys, and humans. Science Translational Medicine.

[35] Coskun T., Sloop K.W., Loghin C., Alsina-Fernandez J., Urva S., Bokvist K.B. (2018). LY3298176, a novel dual GIP and GLP-1 receptor agonist for the treatment of type 2 diabetes mellitus: from discovery to clinical proof of concept. Molecular Metabolism.

[36] Nørregaard P.K., Deryabina M.A., Tofteng Shelton P., Ju Fog, Daugaard J.R., Eriksson P.-O. (2018). A novel GIP analogue, ZP4165, enhances glucagon-like peptide-1-induced body weight loss and improves glycaemic control in rodents. Diabetes, Obesity and Metabolism.

[37] Mroz P.A., Finan B., Gelfanov V., Yang B., Tschöp M.H., DiMarchi R.D. (2019). Optimized GIP analogs promote body weight lowering in mice through GIPR agonism not antagonism. Molecular Metabolism.

[38] Zhang Q., Delessa C.T., Augustin R., Bakhti M., Colldén G., Drucker D.J. (2021). The glucose-dependent insulinotropic polypeptide (GIP) regulates body weight and food intake via CNS-GIPR signaling. Cell Metabolism.

[39] Borner T., Geisler C.E., Fortin S.M., Cosgrove R., Alsina-Fernandez J., Dogra M. (2021). GIP receptor agonism attenuates GLP-1 receptor agonist induced nausea and emesis in preclinical models. Diabetes.

[40] Frías J.P., Bastyr E.J., Vignati L., Tschöp M.H., Schmitt C., Owen K. (2017). The sustained effects of a dual GIP/GLP-1 receptor agonist, NNC0090-2746, in patients with type 2 diabetes. Cell Metabolism.

[41] Frías J.P., Nauck M.A., Van J., Kutner M.E., Cui X., Benson C. (2018). Efficacy and safety of LY3298176, a novel dual GIP and GLP-1 receptor agonist, in patients with type 2 diabetes: a randomised, placebo-controlled and active comparator-controlled phase 2 trial. Lancet.

[42] Frías J.P., Davies M.J., Rosenstock J., Pérez Manghi F.C., Fernández Landó L., Bergman B.K. (2021). Tirzepatide versus semaglutide once weekly in patients with type 2 diabetes. New England Journal of Medicine.

[43] Finan B., Yang B., Ottaway N., Smiley D.L., Ma T., Clemmensen C. (2015). A rationally designed monomeric peptide triagonist corrects obesity and diabetes in rodents. Nature Medicine.

[44] Knerr P.J., Mowery S.A., Finan B., Perez-Tilve D., Tschöp M.H., DiMarchi R.D. (2020). Selection and progression of unimolecular agonists at the GIP, GLP-1, and glucagon receptors as drug candidates. Peptides.

[45] Capozzi M.E., DiMarchi R.D., Tschöp M.H., Finan B., Campbell J.E. (2018). Targeting the incretin/glucagon system with triagonists to treat diabetes. Endocrine Reviews.

[46] Evers A., Pfeiffer-Marek S., Bossart M., Elvert R., Lorenz K., Heubel C. (2020). Multiparameter peptide optimization toward stable triple agonists for the treatment of diabetes and obesity. Advances in Therapy.

[47] Lau J., Bloch P., Schäffer L., Pettersson I., Spetzler J., Kofoed J. (2015). Discovery of the once-weekly glucagon-like peptide-1 (GLP-1) analogue semaglutide. Journal of Medicinal Chemistry.

[48] Ward B.P., Ottaway N.L., Perez-Tilve D., Ma D., Gelfanov V.M., Tschöp M.H. (2013). Peptide lipidation stabilizes structure to enhance biological function. Molecular Metabolism.

[49] Gabery S., Salinas C.G., Paulsen S.J., Ahnfelt-Rønne J., Alanentalo T., Baquero A.F. (2020). Semaglutide lowers body weight in rodents via distributed neural pathways. JCI Insight.

[50] Simonsen L., Lau J., Kruse T., Guo T., McGuire J., Jeppesen J.F. (2022). Preclinical evaluation of a protracted GLP-1/glucagon receptor co-agonist: translational difficulties and pitfalls. PLoS One.

[51] Bossart M., Wagner M., Elvert R., Evers A., Hubschle T., Kloeckener T. (2022). Effects on weight loss and glycemic control with SAR441255, a potent unimolecular peptide GLP-1/GIP/GCG receptor triagonist. Cell Metabolism.

[52] Jiménez A., Mari A., Casamitjana R., Lacy A., Ferrannini E., Vidal J. (2014). GLP-1 and glucose tolerance after sleeve gastrectomy in morbidly obese subjects with type 2 diabetes. Diabetes.

[53] Ahren B., Atkin S.L., Charpentier G., Warren M.L., Wilding J.P.H., Birch S. (2018). Semaglutide induces weight loss in subjects with type 2 diabetes regardless of baseline BMI or gastrointestinal adverse events in the SUSTAIN 1 to 5 trials. Diabetes, Obesity and Metabolism.

[54] Kim T., Nason S., Holleman C., Pepin M., Wilson L., Berryhill T.F. (2018). Glucagon receptor signaling regulates energy metabolism via hepatic farnesoid X receptor and fibroblast growth factor 21. Diabetes.

[55] Nason S.R., Antipenko J., Presedo N., Cunningham S.E., Pierre T.H., Kim T. (2021). Glucagon receptor signaling regulates weight loss via central KLB receptor complexes. JCI Insight.

[56] Dobbs R., Sakurai H., Sasaki H., Faloona G., Valverde I., Baetens D. (1975). Glucagon: role in the hyperglycemia of diabetes mellitus. Science.

[57] Sakurai H., Dobbs R.E., Unger R.H. (1975). The role of glucagon in the pathogenesis of the endogenous hyperglycemia of diabetes mellitus. Metabolism.

[58] Petersen K.M., Bøgevig S., Holst J.J., Knop F.K., Christensen M.B. (2018). Hemodynamic effects of glucagon: a literature review. The Journal of Cinical Endocrinology and Metabolism.

[59] Coskun T., Moyers J.S., Roell W.C., O’Farrell L., Regmi A., Ruan X. (2021). 679-P: the novel GIP, GLP-1, and glucagon triple receptor agonist LY3437943 exhibits robust efficacy in preclinical models of obesity and diabetes. Diabetes.

[60] Blundell J., Finlayson G., Axelsen M., Flint A., Gibbons C., Kvist T. (2017). Effects of once-weekly semaglutide on appetite, energy intake, control of eating, food preference and body weight in subjects with obesity. Diabetes, Obesity and Metabolism.

[61] Willard F.S., Douros J.D., Gabe M.B., Showalter A.D., Wainscott D.B., Suter T.M. (2020). Tirzepatide is an imbalanced and biased dual GIP and GLP-1 receptor agonist. JCI Insight.

[62] Pamir N., Lynn F.C., Buchan A.M.J., Ehses J., Hinke S.A., Pospisilik J.A. (2003). Glucose-dependent insulinotropic polypeptide receptor null mice exhibit compensatory changes in the enteroinsular axis. American Journal of Physiology. Endocrinology and Metabolism.

[63] Ahren B., Yamada Y., Seino Y. (2020). The mediation by GLP-1 receptors of glucagon-induced insulin secretion revisited in GLP-1 receptor knockout mice. Peptides.

[64] Miyawaki K., Yamada Y., Ban N., Ihara Y., Tsukiyama K., Zhou H. (2002). Inhibition of gastric inhibitory polypeptide signaling prevents obesity. Nature Medicine.

[65] Jones B. (2022). The therapeutic potential of GLP-1 receptor biased agonism. British Journal of Pharmacology.

[66] Lundgren J.R., Janus C., Jensen S.B.K., Juhl C.R., Olsen L.M., Christensen R.M. (2021). Healthy weight loss maintenance with exercise, liraglutide, or both combined. New England Journal of Medicine.

[67] Lu S.C., Chen M., Atangan L., Killion E.A., Komorowski R., Cheng Y. (2021). GIPR antagonist antibodies conjugated to GLP-1 peptide are bispecific molecules that decrease weight in obese mice and monkeys. Cell Reports Medicine.

[68] Akbari P., Gilani A., Sosina O., Kosmicki J.A., Khrimian L., Fang Y.Y. (2021). Sequencing of 640,000 exomes identifies GPR75 variants associated with protection from obesity. Science.

[69] Karhunen V., Daghlas I., Zuber V., Vujkovic M., Olsen A.K., Knudsen L.B. (2021). Leveraging human genetic data to investigate the cardiometabolic effects of glucose-dependent insulinotropic polypeptide signalling. Diabetologia.

[70] Baggio L.L., Kim J.-G., Drucker D.J. (2004). Chronic exposure to GLP-1R agonists promotes homologous GLP-1 receptor desensitization in vitro but does not attenuate GLP-1R–dependent glucose homeostasis in vivo. Diabetes.

[71] Urva S., Du Y., Thomas M.K., Milicevic Z., Coskun T., Benson C. (2021). Novel GIP/GLP-1/glucagon receptor agonist LY3437943: a first-in-human dose study in healthy subjects. Diabetes.

[72] Sheahan K.H., Wahlberg E.A., Gilbert M.P. (2020). An overview of GLP-1 agonists and recent cardiovascular outcomes trials. Postgraduate Medical Journal.

[73] Goodwill A.G., Mather K.J., Conteh A.M., Sassoon D.J., Noblet J.N., Tune J.D. (2014). Cardiovascular and hemodynamic effects of glucagon-like peptide-1. Reviews in Endocrine & Metabolic Disorders.

[74] Barragan J.M., Rodrigues R.E., Eng J., Blazquez E. (1996). Interactions of exendin-(9–39) with the effects of glucagon-like peptide-1-[7–36) amide and of exendin-4 on arterial blood pressure and heart rate in rats. Regulatory Peptides.

[75] Winther-Sorensen M., Galsgaard K.D., Santos A., Trammell S.A.J., Sulek K., Kuhre R.E. (2020). Glucagon acutely regulates hepatic amino acid catabolism and the effect may be disturbed by steatosis. Molecular Metabolism.

