# Supplementary material for: Next generation GLP-1/GIP/glucagon triple agonists normalize body weight in obese mice
Source: Mol Metab. 2022 Jul 7;63:101533. doi: 10.1016/j.molmet.2022.101533 (PMC9305623; doi:10.1016/j.molmet.2022.101533)
Supplement: Multimedia component 1 [file mmc1.pptx]

## Slide 1
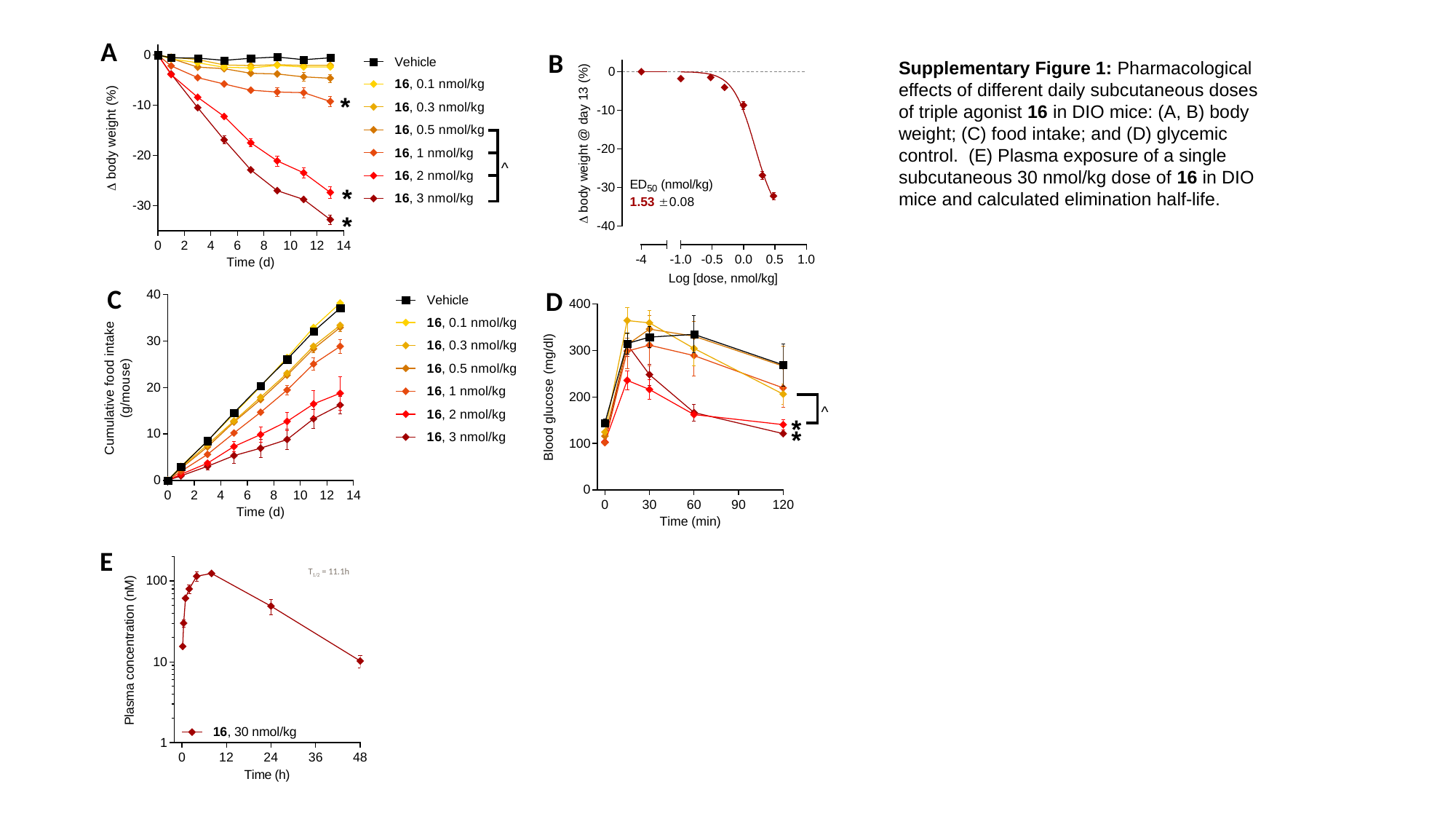

A
B
Supplementary Figure 1: Pharmacological effects of different daily subcutaneous doses of triple agonist 16 in DIO mice: (A, B) body weight; (C) food intake; and (D) glycemic control. (E) Plasma exposure of a single subcutaneous 30 nmol/kg dose of 16 in DIO mice and calculated elimination half-life.
C
D
E
T1/2 = 11.1h

## Slide 2
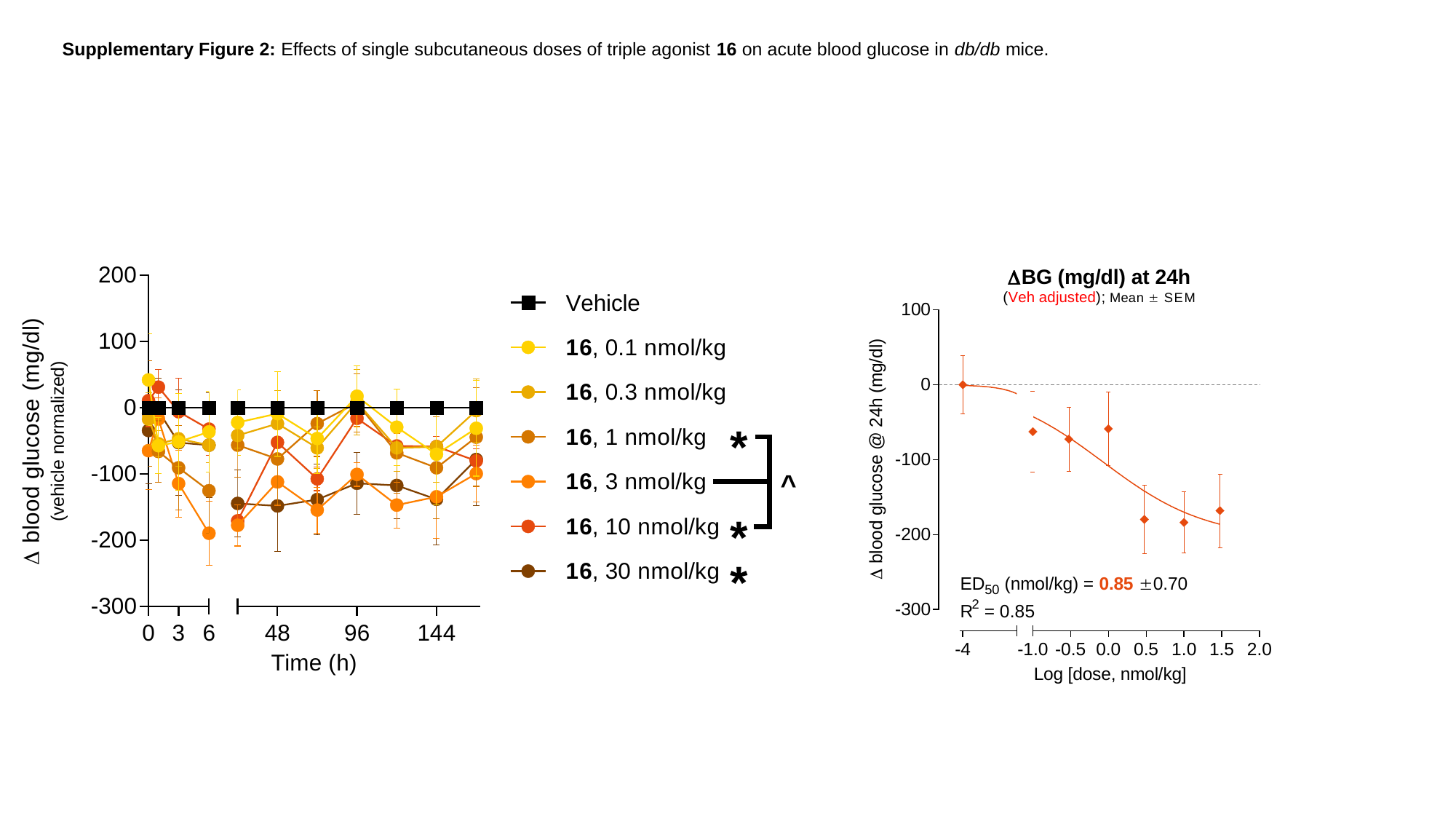

Supplementary Figure 2: Effects of single subcutaneous doses of triple agonist 16 on acute blood glucose in db/db mice.

## Slide 3
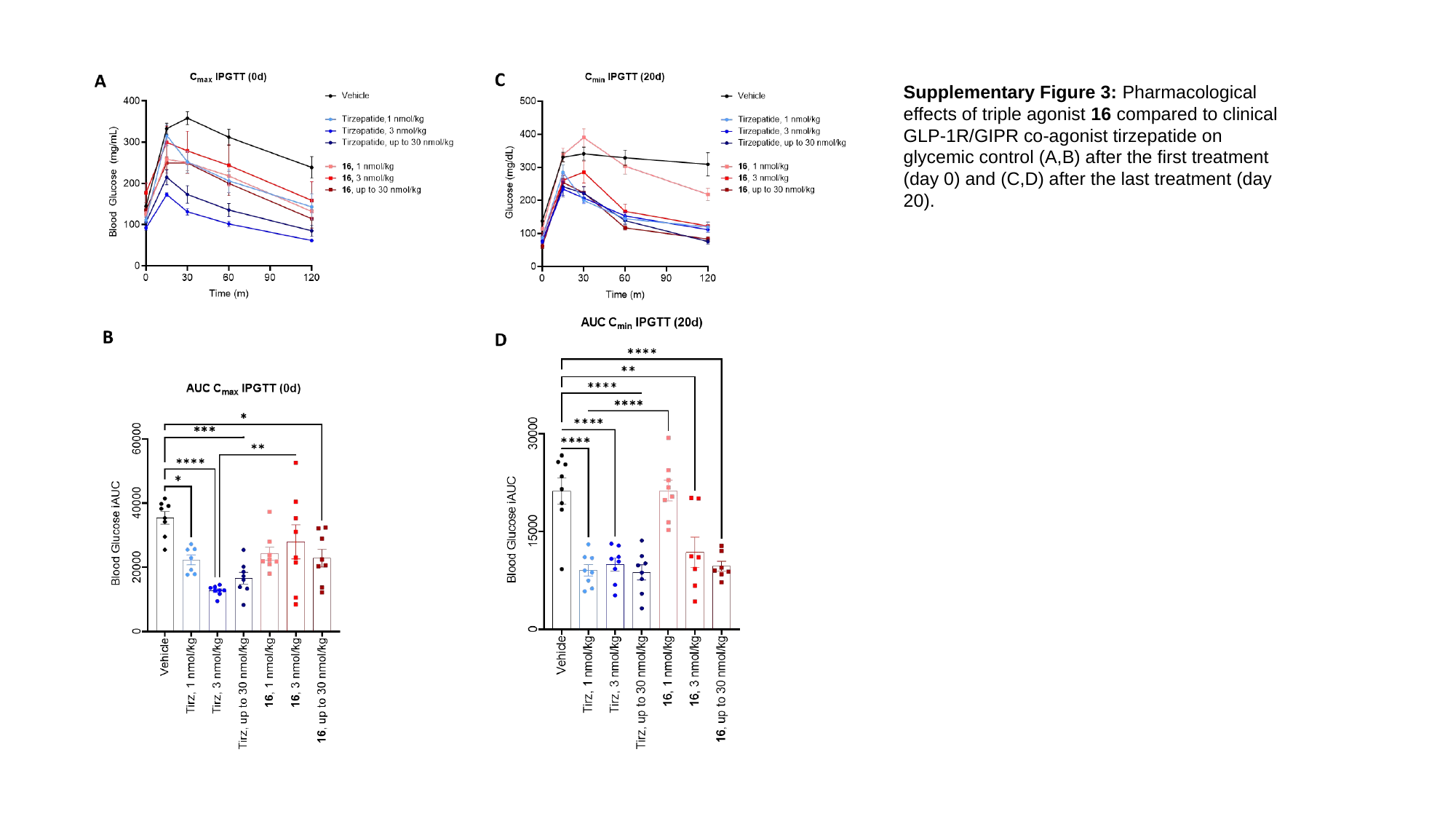

Supplementary Figure 3: Pharmacological effects of triple agonist 16 compared to clinical GLP-1R/GIPR co-agonist tirzepatide on glycemic control (A,B) after the first treatment (day 0) and (C,D) after the last treatment (day 20).

## Slide 4
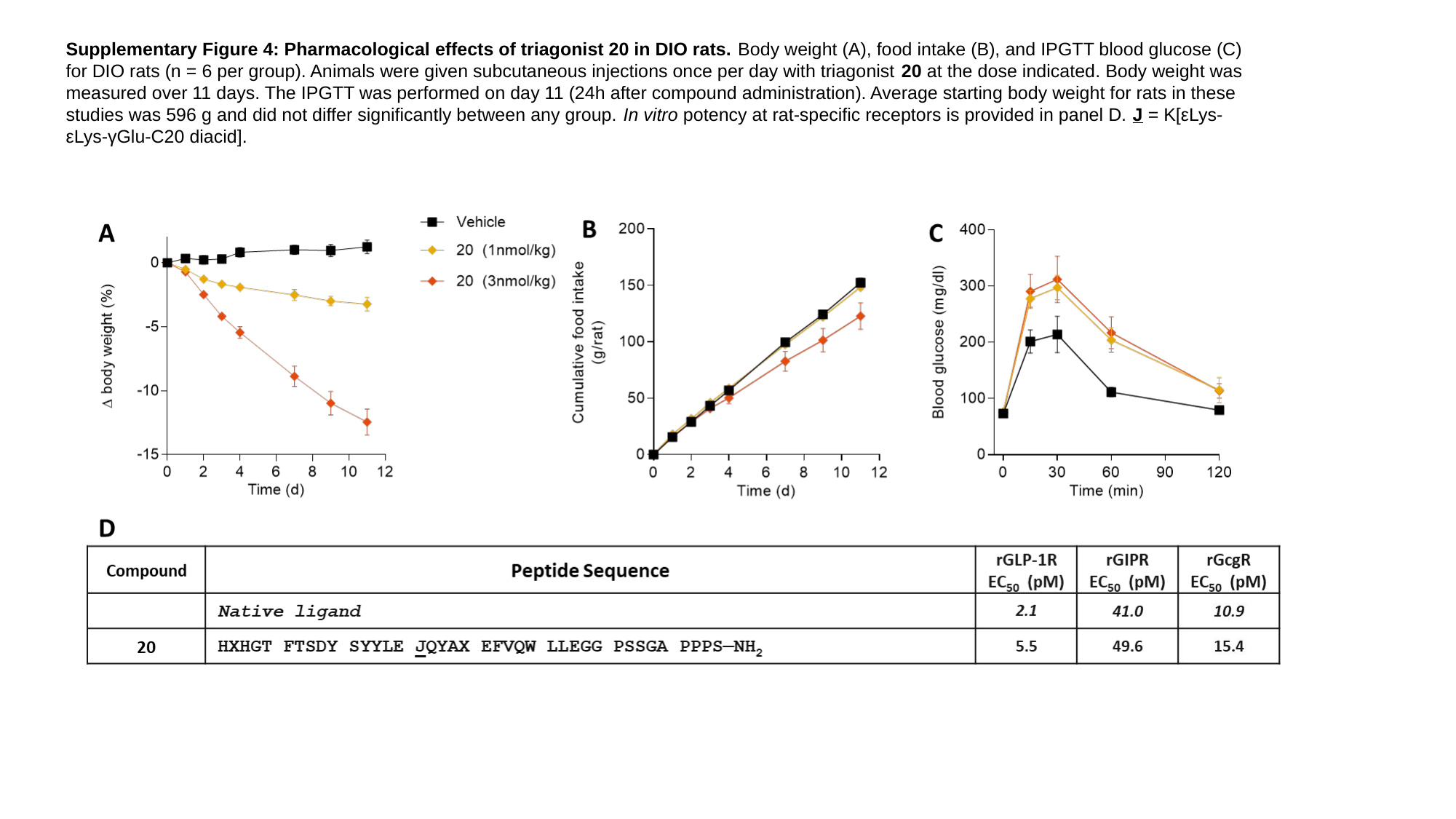

Supplementary Figure 4: Pharmacological effects of triagonist 20 in DIO rats. Body weight (A), food intake (B), and IPGTT blood glucose (C) for DIO rats (n = 6 per group). Animals were given subcutaneous injections once per day with triagonist 20 at the dose indicated. Body weight was measured over 11 days. The IPGTT was performed on day 11 (24h after compound administration). Average starting body weight for rats in these studies was 596 g and did not differ significantly between any group. In vitro potency at rat-specific receptors is provided in panel D. J = K[εLys-εLys-γGlu-C20 diacid].

## Slide 5
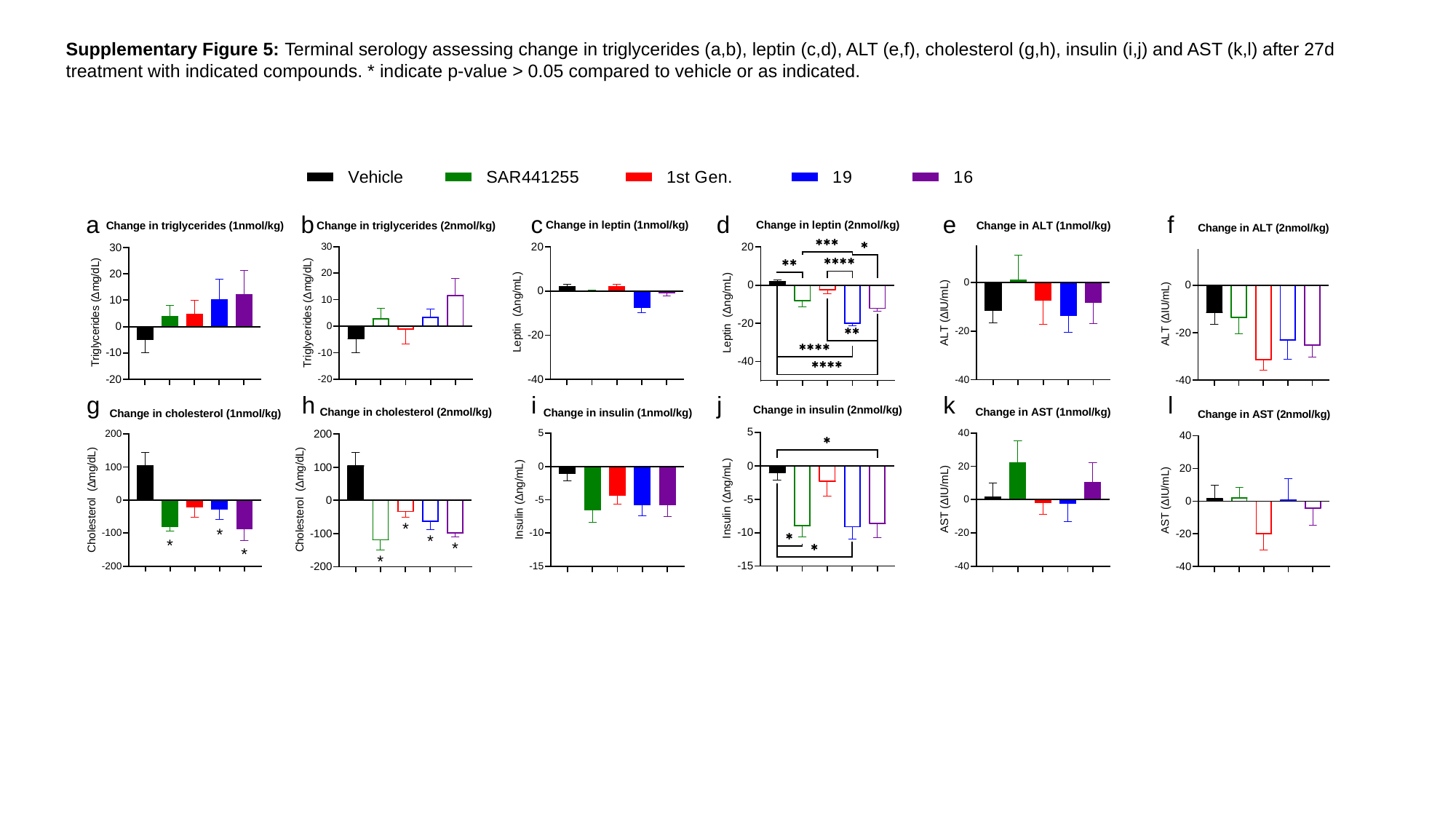

Supplementary Figure 5: Terminal serology assessing change in triglycerides (a,b), leptin (c,d), ALT (e,f), cholesterol (g,h), insulin (i,j) and AST (k,l) after 27d treatment with indicated compounds. * indicate p-value > 0.05 compared to vehicle or as indicated.

## Slide 6
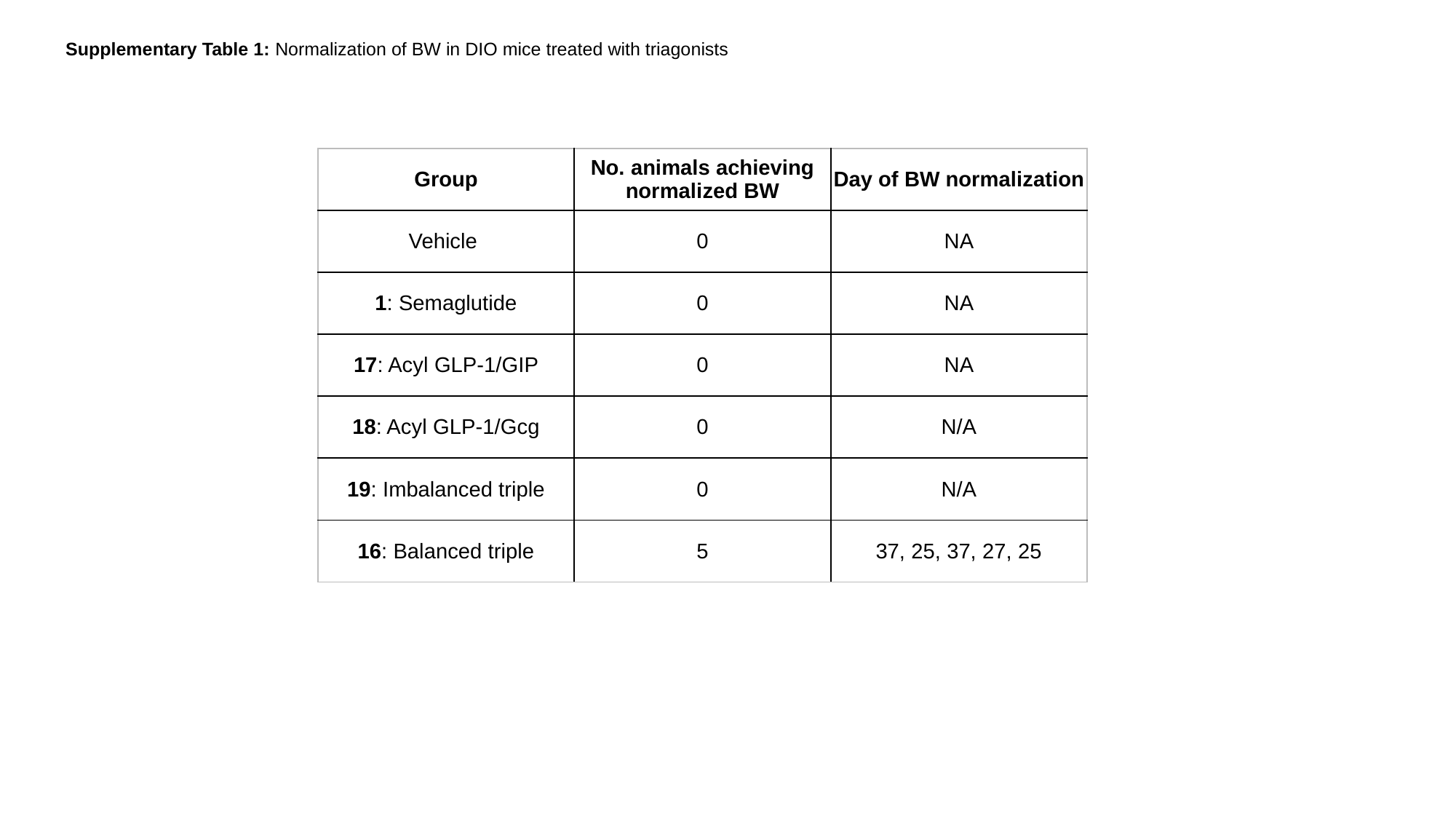

Supplementary Table 1: Normalization of BW in DIO mice treated with triagonists
| Group | No. animals achieving normalized BW | Day of BW normalization |
| --- | --- | --- |
| Vehicle | 0 | NA |
| 1: Semaglutide | 0 | NA |
| 17: Acyl GLP-1/GIP | 0 | NA |
| 18: Acyl GLP-1/Gcg | 0 | N/A |
| 19: Imbalanced triple | 0 | N/A |
| 16: Balanced triple | 5 | 37, 25, 37, 27, 25 |

## Slide 7
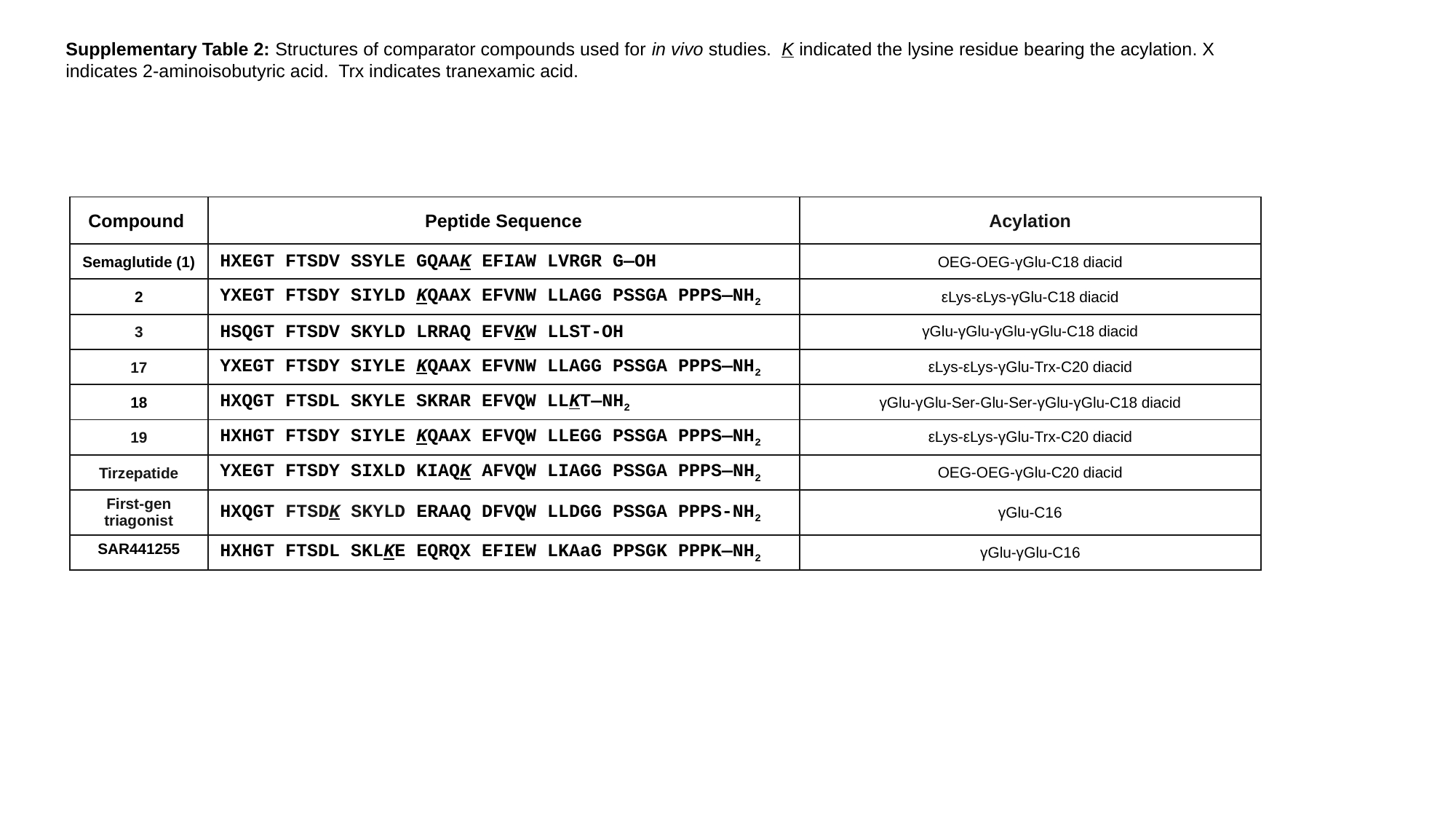

Supplementary Table 2: Structures of comparator compounds used for in vivo studies.  K indicated the lysine residue bearing the acylation. X indicates 2-aminoisobutyric acid.  Trx indicates tranexamic acid.
| Compound | Peptide Sequence | Acylation |
| --- | --- | --- |
| Semaglutide (1) | HXEGT FTSDV SSYLE GQAAK EFIAW LVRGR G—OH | OEG-OEG-γGlu-C18 diacid |
| 2 | YXEGT FTSDY SIYLD KQAAX EFVNW LLAGG PSSGA PPPS—NH2 | εLys-εLys-γGlu-C18 diacid |
| 3 | HSQGT FTSDV SKYLD LRRAQ EFVKW LLST-OH | γGlu-γGlu-γGlu-γGlu-C18 diacid |
| 17 | YXEGT FTSDY SIYLE KQAAX EFVNW LLAGG PSSGA PPPS—NH2 | εLys-εLys-γGlu-Trx-C20 diacid |
| 18 | HXQGT FTSDL SKYLE SKRAR EFVQW LLKT—NH2 | γGlu-γGlu-Ser-Glu-Ser-γGlu-γGlu-C18 diacid |
| 19 | HXHGT FTSDY SIYLE KQAAX EFVQW LLEGG PSSGA PPPS—NH2 | εLys-εLys-γGlu-Trx-C20 diacid |
| Tirzepatide | YXEGT FTSDY SIXLD KIAQK AFVQW LIAGG PSSGA PPPS—NH2 | OEG-OEG-γGlu-C20 diacid |
| First-gen triagonist​ | HXQGT FTSDK SKYLD ERAAQ DFVQW LLDGG PSSGA PPPS-NH2 | γGlu-C16 |
| SAR441255​ | HXHGT FTSDL SKLKE EQRQX EFIEW LKAaG PPSGK PPPK—NH2 | γGlu-γGlu-C16 |
